# Overview and current status of published research on cancer, sarcopenia and physical activity: A bibliometric analysis

**DOI:** 10.3934/publichealth.2025033

**Published:** 2025-06-23

**Authors:** J. A. Parraca, D. Salas-Gómez, R. Dinis, A. Denche-Zamorano, A. Vega-Muñoz, P. Tomas-Carus

**Affiliations:** 1 Departamento de Desporto e Saúde, Escola de Saúde e Desenvolvimento Humano, Universidade de Évora, 7004-516 Evora, Portugal; 2 Comprehensive Health Research Centre (CHRC), University of Evora, 7004-516 Evora, Portugal; 3 Medical Oncology, Hospital do Espirito Santo de Evora EPE, Evora, Portugal; 4 Promoting a Healthy Society Research Group (PHeSO), Faculty of Sport Sciences, University of Extremadura, 10003 Caceres, Spain; 5 Centro de Investigación en Educación de Calidad para la Equidad, Universidad Central de Chile, Santiago 8330601, Chile; 6 Facultad de Ciencias Empresariales, Universidad Arturo Prat, Iquique 1110939, Chile

**Keywords:** physical inactivity, review, neoplasia, physical function, loss mass, quality of life

## Abstract

**Introduction:**

Cancer is a global health problem; the presence of secondary symptoms such as sarcopenia in cancer patients is relatively common. Physical activity (PA) is notable for its protective role against sarcopenia; however, there is currently no bibliometric analysis of research related to cancer, sarcopenia, and physical activity.

**Methods:**

A search on the Web of Science (WoS) Core Collection database was performed on this topic, and a bibliometric analysis of the identified publications was performed using traditional bibliometric laws.

**Results:**

121 publications were found. Annual publications presented an exponentially growing trend from 2012 to 2023 (*R^2^* = 91%). The United States of America was the country with the most documents worldwide. Newton, R. U. and Galvao, D. A. were highlighted as the most prolific and prominent co-authors. The Cancers and Journal of Cachexia, Sarcopenia and Muscle were the journals with the highest number of published documents. 36 papers, having 37 or more citations, were the most cited papers. The author keywords and keywords Plus® identified thematic clusters related to research on this topic, such as cancer, physical activity, aging, muscle, skeletal muscle, osteoporosis, malnutrition, body composition, cachexia, survival, and frailty.

**Conclusion:**

Research on cancer, sarcopenia, and physical activity has followed an exponential growth trend, which reveals growing interest in the topic. Significant authors and collaborative groups in the field were identified, as well as the journals and countries with the highest number of publications and the research trends most followed by researchers.

## 1. Introduction

Cancer is a global health problem, and it is believed that around 1 in 5 people will develop some form of cancer in their lifetime [1]. According to the World Health Organization (WHO), cancer is responsible for approximately 1 in 6 deaths worldwide [2]. The most common types of cancer include breast, lung, colon and rectum, prostate, skin, and stomach cancer [3].

Although cancer remains a major cause of death, life expectancy and the number of survivors have significantly increased thanks to advances in treatment and early detection [4]–[7]. However, this implies new challenges for public and community health, as the process a cancer patient goes through and the side effects of treatments have a significant impact on the quality of life of survivors [8].

Side effects that may occur during or after treatment, even delayed and persisting throughout life, include dental problems, early menopause, fatigue, heart problems, risk of developing other cancers, cognitive problems, depression, anxiety, osteoporosis, changes in body weight, and impaired overall physical functioning [7],[9]–[11].

Weight loss after cancer treatment is common and may be related to insufficient protein and calorie intake, which can lead to sarcopenia [11]–[13]. The presence of sarcopenia in cancer patients is a relatively common symptom, with a prevalence ranging from 14% to 79% [14]. A large number of women with breast cancer, for example, experience a loss of lean mass and an increase in fat mass after therapy, which can lead to the presence of sarcopenia or sarcopenic obesity [15]–[17].

Sarcopenia is characterized by loss of muscle mass and decreased strength and physical performance, and is diagnosed when the appendicular skeletal muscle mass index is significantly below the sex-age adjusted mean [18]. Sarcopenia represents one of the greatest health challenges and is frequently associated with falls, frailty, and physical and functional limitations, making daily activities difficult [13],[18]. The causes of sarcopenia are multifactorial and include the loss of alpha motor neurons, the secretion of pro-inflammatory factors such as interleukin 6, or the inhibition of growth hormone secretion [19]. Related to cancer, sarcopenia is also a risk factor for mortality and increased risk of chemotherapy toxicity [11],[13],[20]. In this context, it has been reported that women with breast cancer and sarcopenia have a worse prognosis and an increased risk of mortality [21],[22]. Poorer prognostic outcomes or increased post-operative complications have also been reported in lung cancer patients [23],[24]. In addition, a recent systematic review suggests that sarcopenia may be a risk factor for reduced progression-free survival in patients with metastatic cancer [25].

In addition to these factors, there are other factors associated with treatment, such as malnutrition and physical inactivity in cancer patients [7],[12].

Physical inactivity is a major risk factor for mortality worldwide, as well as for the development of many chronic diseases, including cancer [7],[26]. One study found that physical inactivity was responsible for 2.9% of all cancers [27]. In this regard, it is important to note that sarcopenia related to cancer decreased physical performance in these patients [13]. For these reasons, targeting physical inactivity in cancer patients is a priority because of its negative impact, both as a risk factor for the disease itself and because of its contribution to the development of sarcopenia. This makes physical inactivity one of the main modifiable risk factors for poor prognosis and poor treatment outcomes in these patients [28].

In contrast, physical activity (PA) is notable for its protective role against sarcopenia [7]. In particular, PA and exercise prevent muscle degradation by restoring redox homeostasis through increased production of antioxidant and anti-inflammatory substances. In addition, PA helps to increase lean mass and muscular endurance and improve the functionality of individuals [7],[29]. Current evidence suggests that cancer survivors can safely engage in PA and physical training to improve fitness, restore physical functioning, improve bone mass, improve quality of life, and mitigate cancer-related fatigue [30]. However, there is currently insufficient evidence to make specific physical activity recommendations for each secondary symptom in cancer survivors [7].

Therefore, the same recommendations are followed as for people with chronic diseases: at least 150 minutes per week of aerobic activity, two or more days of resistance training, and daily stretching, adjusted according to health status and side effects of treatment [7]. In this sense, the World Health Organization defines physical activity as any bodily movement produced by skeletal muscles that requires energy expenditure [7].

Addressing sarcopenia in cancer patients or cancer survivors is a health challenge that has attracted increasing global interest among both researchers and clinicians. A recent bibliometric analysis reported an exponential growth in the study of cancer and sarcopenia, with an *R²* of 70%, reflecting a large critical mass of research in this field [31]. This publication highlights the ten most cited articles related to cancer-associated sarcopenia research that mainly focus on the association between different types of cancer with factors such as cancer-associated malnutrition and body mass index [31]. However, there is currently no bibliometric analysis of research related to cancer, sarcopenia, and physical activity.

Therefore, the aim of this study was to analyze this topic in depth through a bibliometric analysis, as well as uncover new issues of interest to researchers in this area, verify the annual trend of publications, identify the categories, journals, and countries with the highest number of publications on the topic, point out the most productive and prominent authors, highlight the most cited articles, and detail how many and which keywords are most used by the authors. Bibliometric analysis offers a more objective perspective on the current state of research compared to traditional literature reviews. Traditional reviews can include subjective assessments, leading to some variability, whereas bibliometric analysis relies on statistical and mathematical analysis [32].

In this way, the following hypotheses were established: 1) Research related to sarcopenia, physical activity, and cancer is in a phase of exponential growth. 2) A small number of journals (nucleus of journals) and authors (prolific authors) concentrate the largest number of publications on the topic. 3) There are a small number of keywords that authors use more frequently than others in publications on this topic.

## 2. Materials and methods

A bibliometric analysis was carried out based on the traditional laws of bibliometrics, including data from articles published in journals indexed in the Science Citation Index Expanded (SCI-E), Social Science Citation Index (SSCI), and Emerging Sources Citation Index (ESCI) databases of the main collection of the Web of Science [33].

The following search vector was used to obtain the set of documents: (TI = (cancer AND sarcopeni* AND (physical NEAR/0 activity)) OR (AB = (cancer AND sarcopeni* AND (physical NEAR/0 activity)) OR (AK = (cancer AND sarcopeni* AND (physical NEAR/0 activity))). The search vector included the tags TI (search in title), AB (search in abstract), and AK (search in author keyword), the asterisk ‘*’ (search for terms with the root sarcopeni), and the textual proximal connector ‘NEAR/0’ (search for terms appearing together). Furthermore, the search was limited to articles and reviews. No other limitations, such as years of publication or language exclusions, were applied. The search was conducted on September 16, 2024, by researchers D.S.-G. and A.D.-Z. to ensure that the same set of articles was obtained. The document set was extracted in plain text and .xls (Microsoft Excel).

The authors D.S.-G. and A.D.-Z. reviewed all papers for suitability, applying the following selection criteria: 1) Meeting the search requirements. 2) Human research.

The publication years of the resulting set of papers were analyzed, analyzing the trend followed by the annual publications. The price exponential growth law was applied to check whether the topic is currently in a phase of exponential growth [34],[35]. Exponential growth of an object of study indicates a growing interest among researchers and a large critical mass for its development. For this purpose, the *R^2^* fit of the trend of annual publications to an exponential growth rate was evaluated [36]. A descriptive analysis of the WoS subject categories to which the documents were related was carried out, checking the categories with the highest number of publications. The Hirsch Index (h-index) was used to identify the most cited documents. To do this, all the documents were ordered from the highest to the lowest number of citations, identifying as the most cited documents the h documents with h or more citations [37]–[39]. Bradford's law was applied to check the concentration of documents by journals and to identify the most specialized journals in the subject [40]–[42]. A descriptive analysis of the journals that made up the publication core was carried out, including journal name, publisher, documents, citations, normalized citations (citations/documents), journal impact factor (JIF), quartile, and percentage of Gold Open Access. The authors of all papers were analyzed, checking the number of papers per author. To identify prolific authors, Lotka's law was applied, and a co-authorship analysis was performed with the bibliometric software VOSviewer to identify possible clusters of production among prolific authors [43]–[46]. Prolific authors with one or more highly cited papers (in h-index subset) were checked to identify prominent authors (prolific authors with one or more highly cited papers) [42],[47]–[49].

A descriptive analysis of prominent authors was performed, showing name, country, number of papers, citations, top-cited papers, and production cluster to which they were found to be related. A descriptive co-authorship analysis was performed with the regions/countries, checking which had the highest number of papers and citations. A co-authorship analysis was performed in VOSwiewer to produce a graph showing the international production networks [50]. Finally, Zipf's law was applied to identify the author keywords and Keywords Plus® with the highest co-occurrence in the set of documents [42],[51],[52]. Co-occurrence analyses were carried out in VOSviewer with the most co-occurring keywords and Keywords Plus® to identify possible thematic clusters and the average year of publication for each of the terms [50].

## 3. Results

The WoS search yielded 129 papers, although only 121 met the search and selection criteria (8 animal-based research papers were excluded). Figure S1 shows the flowchart of how the final set of documents was obtained. Therefore, the final sample presented 121 documents: 80 articles (66.4%) and 41 reviews (33.6%).

### 3.1. Publication trends

**Figure 1.**
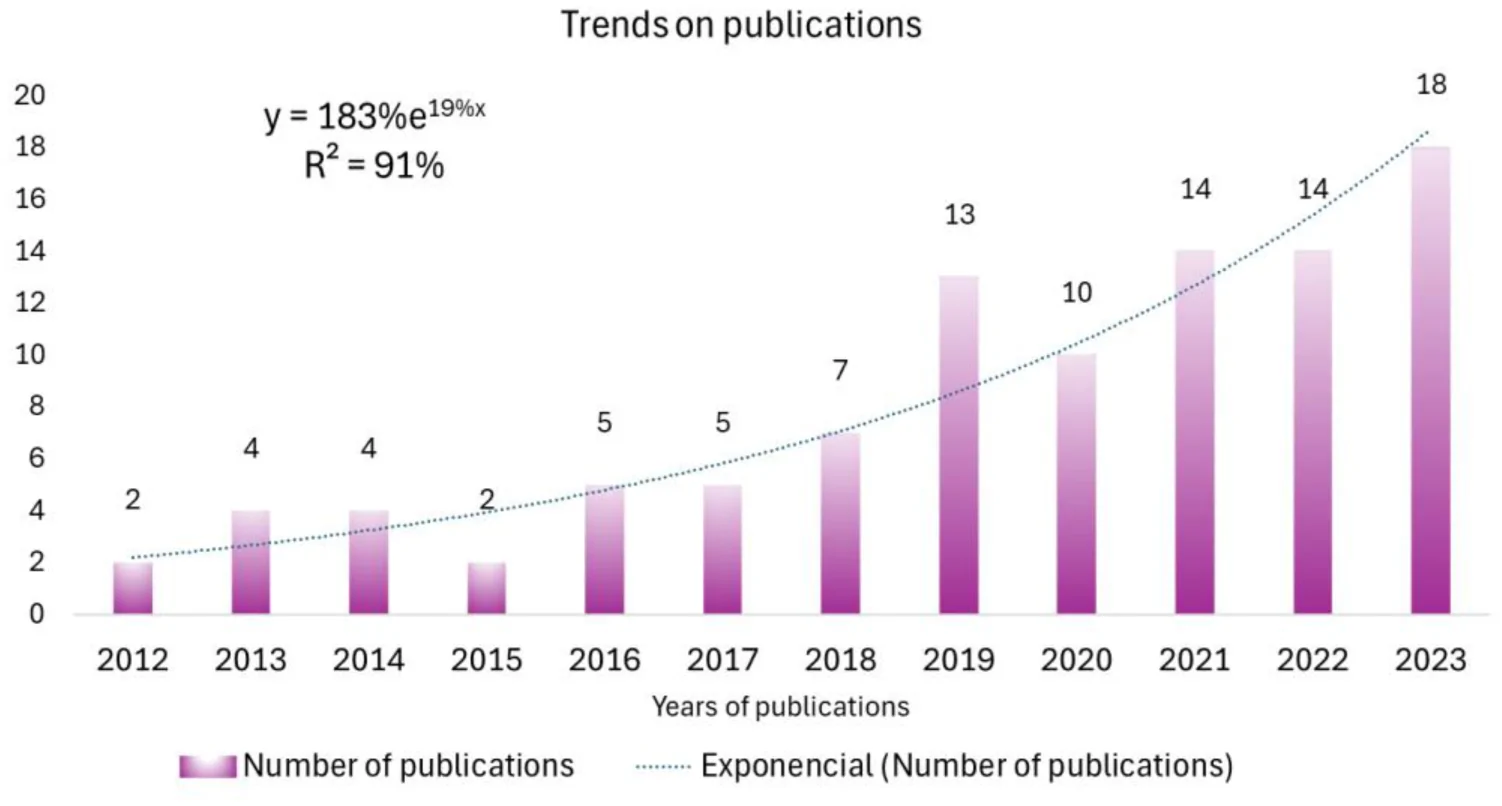
Annual publications trend. The bars represent the frequency of publications in each year (x-axis). The blue line is an exponential trend line.

The first publications indexed in WoS date from the year 2000. Since the first publication, there has been an irregular growth in annual publications until a stabilization from 2012 forward. In the years prior to 2012, there was continuity in the yearly publications between 2000 and 2004 and 2007 and 2009. In all these years, the year with the highest number of yearly publications was 2003, with three publications. However, after this first period, in which publications and pioneering authors of the subject appeared, a growth in publications began in 2012, which has continued up to the present day. In these years, the subject of study entered the phase of exponential growth, which it is still currently experiencing (*R^2^* = 91%). Research related to cancer, sarcopenia, and physical activity has been advancing so quickly that 64% of the total publications have been published from 2019 to the present (78 papers). Figure 1 shows the trend followed by annual publications.

### 3.2. WoS categories

The object of study has been addressed in different areas. Publications were found to be related to 36 WoS subject categories. The category with the highest number of publications was oncology (36 documents), with double the number of publications of the next highest category, nutrition dietetics (17 documents). The other categories with the highest number of related documents were: geriatrics, gerontology, and general internal medicine (13 documents), endocrinology metabolism (11 documents), and healthcare sciences services (8 documents). Table 1 shows the top five thematic categories in the Web of Science, according to the number of documents in which publications are indexed.

**Table 1.** Top five thematic categories in the Web of Science, according to the number of documents in which publications are indexed.

**WoS categories**	**Docs.**	**Main journals**	**Docs.**	**Main publishers**	**Docs.**
Oncology	36	*Cancers*	5	MDPI	5
Nutrition dietetics	17	*Current opinion in clinical nutrition and metabolic care*	4	Springer Nature	5
Geriatrics gerontology	13	*Journal of cachexia sarcopenia and muscle*	5	Wiley	6
Medicine general internal	13	*Journal of cachexia sarcopenia and muscle*	5	Wiley	6
Endocrinology metabolism	11	*Current opinion in clinical nutrition and metabolic care*	5	Lippincott Williams & Wilkins	4

Note: WoS, Web of Science); Docs, documents; Main journals, journals with more documents in each WoS category; Main publishers, publishers with more documents in each WoS category.

### 3.3. Most cited documents

**Figure 2.**
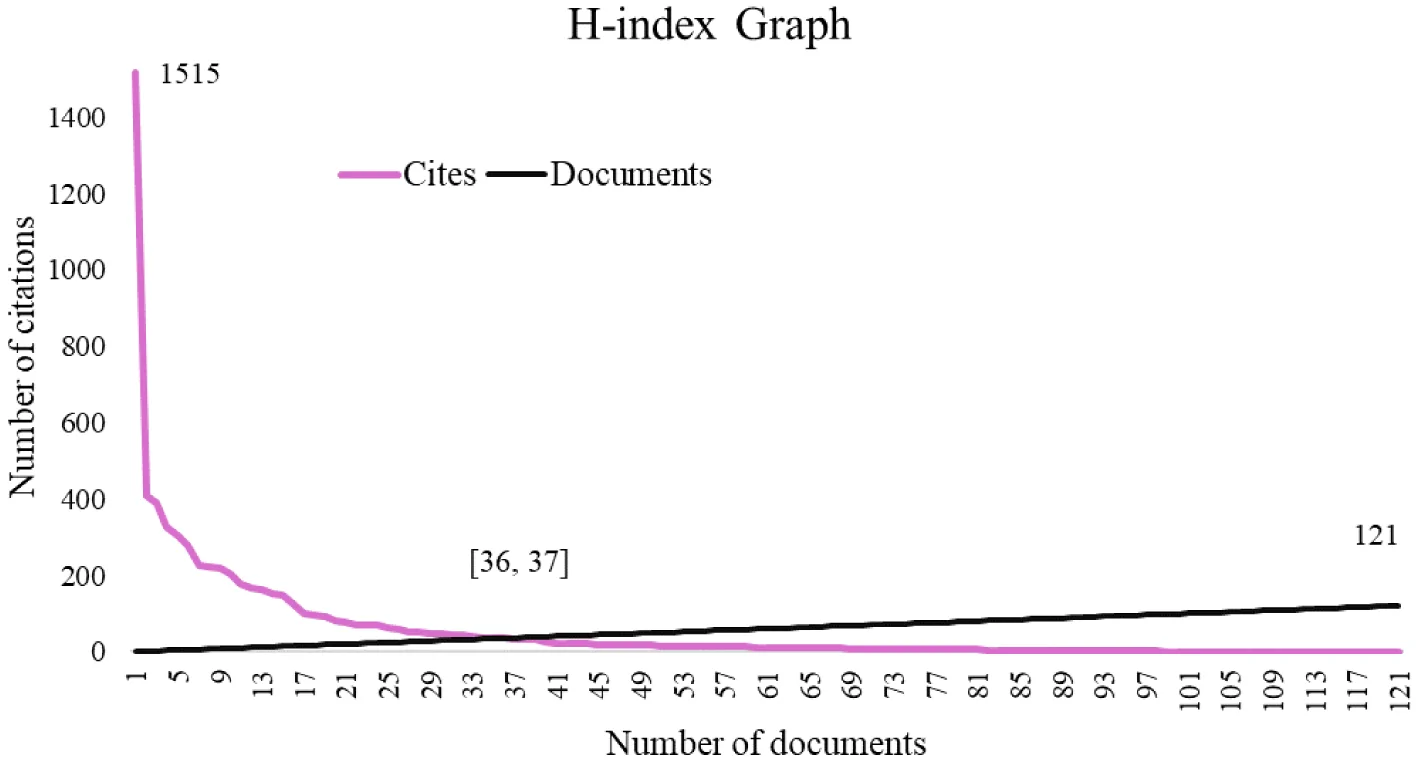
H-index graph.

The range of citations presented by the analyzed papers was between 0 and 1515 citations. There was one paper that stood out above the rest in terms of citations, having three times more citations than the next most cited paper (409 citations). This was an article published in the journal *Comprehensive Physiology* by Booth et al, [53] accumulating 1515 citations. These two top-cited papers were among the 36 papers with 37 or more citations that made up the set of most-cited papers in the subject area. These 36 most cited papers are shown in Supplementary Table S1, all of which had a citation level well above the median for all publications (13 citations). Figure 2 shows a graphical representation of the H-index.

### 3.4. Nucleus of journals

In terms of the number of publications, *Cancers* (MDPI) and *Journal of Cachexia, Sarcopenia and Muscle* (Wiley) were the most prominent journals, both with 5 papers. The core of journals that concentrated the first tercile of publications was composed of 15 journals (Table 2). These journals published 33.1% of the papers with a publication rank between 2 and 5, accounting for only 15.6% of the total journals (96 journals). Although a core of journals could be established by applying Bradford's law, due to the high peripheral dispersion of the journals in terms of their number of publications, the journals could not be segmented into the three classical Bradford zones. Therefore, in addition to the core of journals, a large zone 1 was formed, consisting of 81 journals with a total of 81 papers (66.9%), all with only one paper related to the topic. In other words, 66.9% of the papers were published in journals with a single publication in the object of study of this research.

Of the 36 most cited papers, only 14 were published in journals belonging to the publication core; the remaining 23 were published in journals with only one paper related to the topic. In contrast, journals such as *Frontiers in Physiology*, *Current Opinion in Clinical Nutrition and Metabolic Care*, and *Journal of Clinical Oncology* had more than one highly cited paper.

### 3.5. Prolific and prominent authors

The papers analyzed in this research were the result of the collaboration of 761 authors. Applying Lotka's law, it was estimated that the prolific authors should be the 27 with the highest number of papers. We found that 95.7% of authors submitted a single paper (728 authors), while only 1.2% of authors submitted 3 or more papers (9 authors). When 33 authors with 2 or more papers were found, they were the prolific authors of the subject.

**Figure 3.**
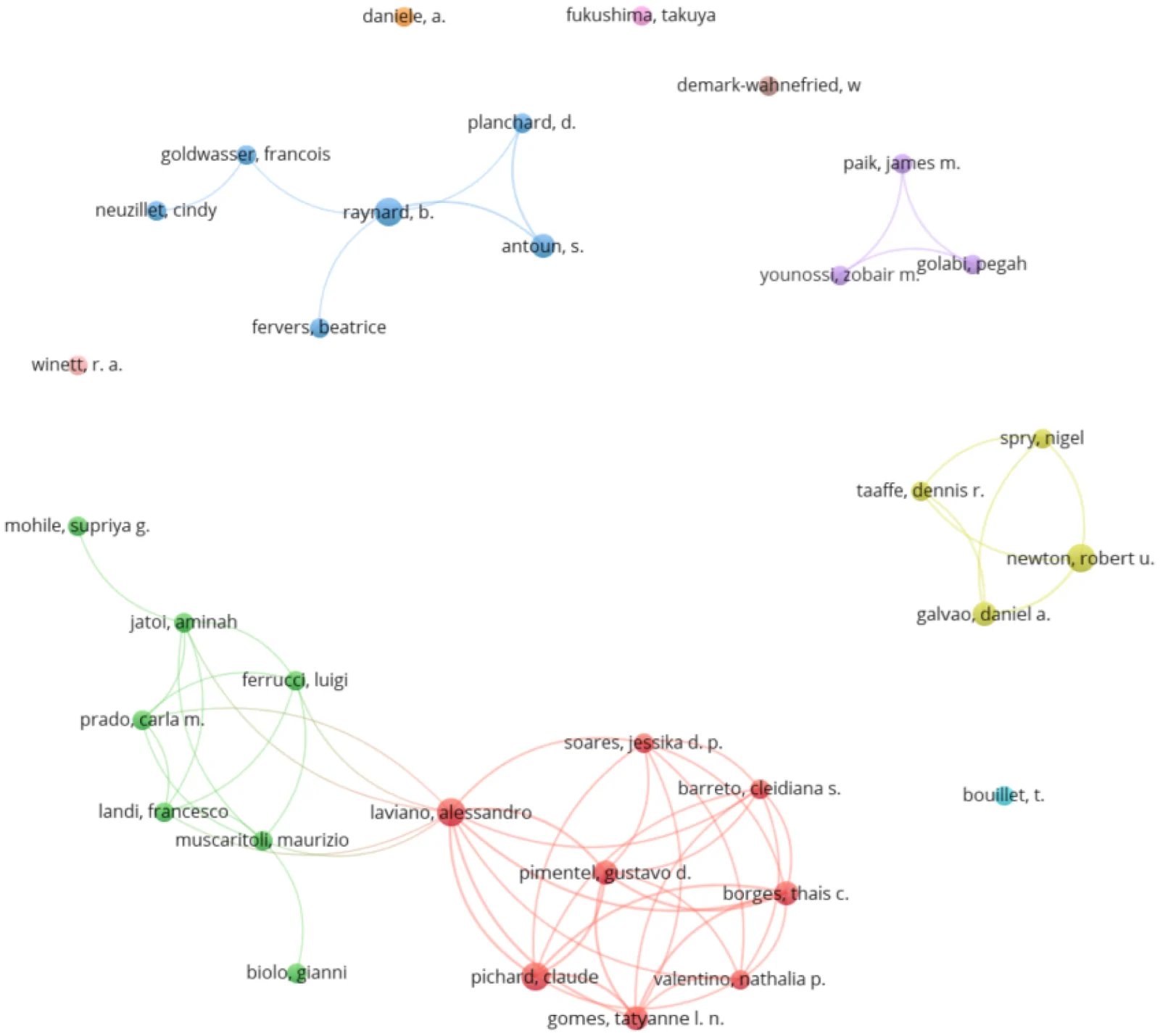
Prolific co-authorship graph.

Among the prolific authors, by level of production, four authors stood out with four papers published on the subject: A. Laviano, R. U. Newton, C. Pichard, and B. Raynard. Laviano and Pichard were part of the most numerous production cluster with a total of 8 prolific authors (Red Cluster), being also the production cluster with the most recent average year of publication. This production cluster was linked, through A. Laviano, with the second most numerous production cluster, formed by 7 authors (Green Cluster), with authors such as L. Ferruci, F. Landi, or F. Muscaritoli. The two most prominent authors in terms of production level, R. Newton and B. Raynard, also appeared at the top of the list. Raynard also appeared leading in terms of production and production clusters. Newton was part of a production group composed of 6 prolific authors (Blue Cluster), while Raynard appeared in a small cluster with four co-authors (Yellow Cluster). Figure 3 shows the 33 prolific authors and the collaborative networks formed by them. Figure S2 shows the same graph, but showing the co-authors according to the average year of their publications.

**Table 2.** Nucleus of journals.

**Publication titles (publisher)**	**Doc.**	**Cit.**	**Norm. Cit.**	**JIF**	**Quartile**	**% O.A.**
*Cancers* (MDPI)	5	161	32	4.5	Q1	96.6
*Journal of Cachexia, Sarcopenia and Muscle* (Wiley)	5	473	95	9.4	Q1	72.2
*Current Opinion in Clinical Nutrition and Metabolic Care* (Lippincott Williams & Wilkins)	4	238	60	3.0	Q2	8.3
*European Journal of Clinical Nutrition* (Springer)	3	346	115	3.6	Q2	24.3
*Frontiers in Physiology* (Frontiers Media)	3	593	198	3.2	Q2	99.5
*Breast Cancer Research and Treatment* (Springer)	2	29	15	3.0	Q2	31.8
*Bulletin du Cancer* (Elsevier)	2	3	2	1.1	Q4	28.0
*Cancer* (Wiley)	2	112	56	6.1	Q1	20.1
*Clinical Nutrition* (Churchill Livingstone)	2	291	146	6.6	Q1	30.3
*Current Oncology* (MDPI)	2	14	7	2.8	Q2	99.5
*International Journal of Environmental Research and Public Health*	2	31	16	n.a.	n.a.	96.1
*Journal of Clinical Oncology* (Lippincott Williams & Wilkins)	2	437	219	42.1	Q1	30.0
*Journal of Personalized Medicine* (MDPI)	2	19	10	3.0	Q1	99.7
*Nutrients* (MDPI)	2	36	18	4.8	Q1	99.1
*Supportive Care in Cancer* (Springer)	2	57	29	2.8	Q1	25.8

Note: Doc., number of documents; Cit., citations; Norm. Cit., normalized citations (citations/documents); JIF, journal impact factor; Quartile, JIF quartile; % O.A., percentage of Gold Open Access; n.a., not applicable.

Cross-checking the prolific authors with the authorships of the most cited papers, 22 prominent authors were found. To be considered a prominent author, it was necessary to have published 2 or more papers and to have one or more papers among the most cited. In this sense, authors such as R. U. Newton (4 documents, 331 citations, 2 most cited papers) and D. A. Galvao (3 documents, 317 citations, 2 most cited papers) stood out. Table 3 shows these prominent authors.

**Table 3.** Prominent authors.

**Authors**	**Country**	**Cluster**	**Doc.**	**Cit.**	**h-index papers**
Newton, R. U.	Australia	Yellow	4	331	2
Galvao, D. A.	Australia	Yellow	3	317	2
Muscaritoli, M.	Italy	Green	2	689	2
Landi, F.	Italy	Green	2	635	2
Ferrucci, L.	USA	Green	2	629	2
Jatoi, A.	USA	Green	2	538	2
Biolo, G.	Italy	Green	2	357	2
Winett, R. A.	USA	n.a.	2	224	2
Mohile, S. G.	USA	Green	2	223	2
Laviano, A.	Italy	Red	4	429	1
Prado, C. M.	Canada	Green	2	410	1
Demark-Wahnefried, W.	USA	n.a.	2	407	1
Spry, N.	Australia	Yellow	2	217	1
Taaffe, D. R.	Australia	Yellow	2	217	1
Goldwasser, F.	France	Blue	2	113	1
Neuzillet, C.	France	Blue	2	99	1
Pichard, C.	Switzerland	Red	4	92	1
Golabi, P.	USA	Purple	2	77	1
Paik, J. M.	USA	Purple	2	77	1
Younossi, Z. M.	USA	Purple	2	77	1
Raynard, B.	France	Blue	4	61	1
Antoun, S.	France	Blue	3	60	1

Note: Doc., number of documents; Cit., Citations.

### 3.6. Countries/regions co-authorship

**Figure 4.**
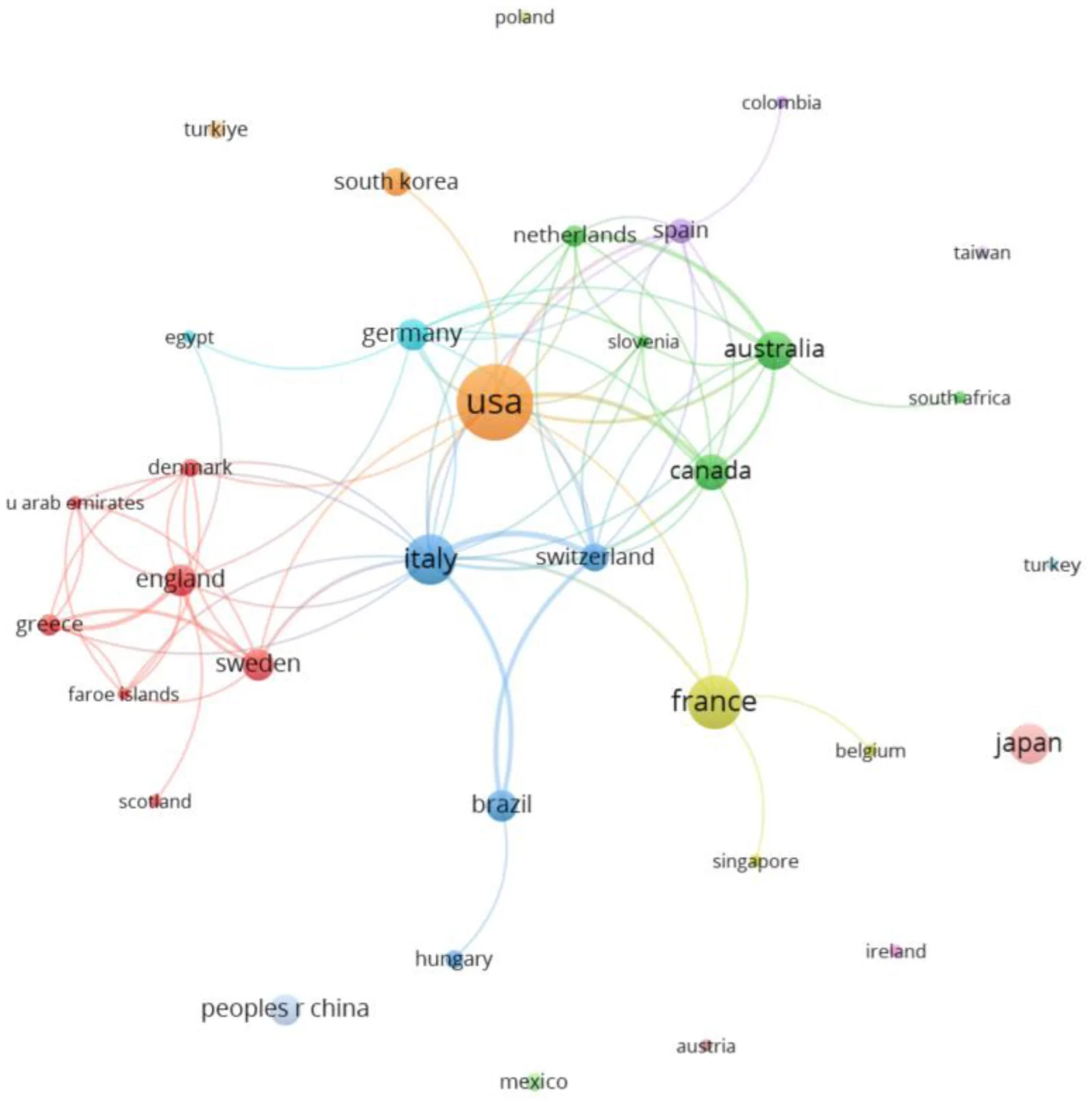
Countries/Regions co-authorship graph.

With a range of publications between 1 and 36 papers, 34 countries/regions were found in co-authorship. The USA was the most prominent country in terms of the number of publications (38 documents), doubling the output of the next most productive country, France (18 documents). Alongside these two countries, others such as Italy (16 documents), Japan (10 documents), and Australia (9 documents) also stood out. In number of citations, the USA was also the most cited country (4528 citations), tripling the following countries, Italy and Denmark, both with 1524 citations. Figure 4 shows the global production network. Although the USA was the most productive country, Italy was the country with the highest number of links to other countries (16 links), followed by the USA (12 links). On the other hand, the most numerous production network was found around England, formed by 7 countries/regions (Cluster Network).

### 3.7. Author's keywords and Keywords Plus®

**Figure 5.**
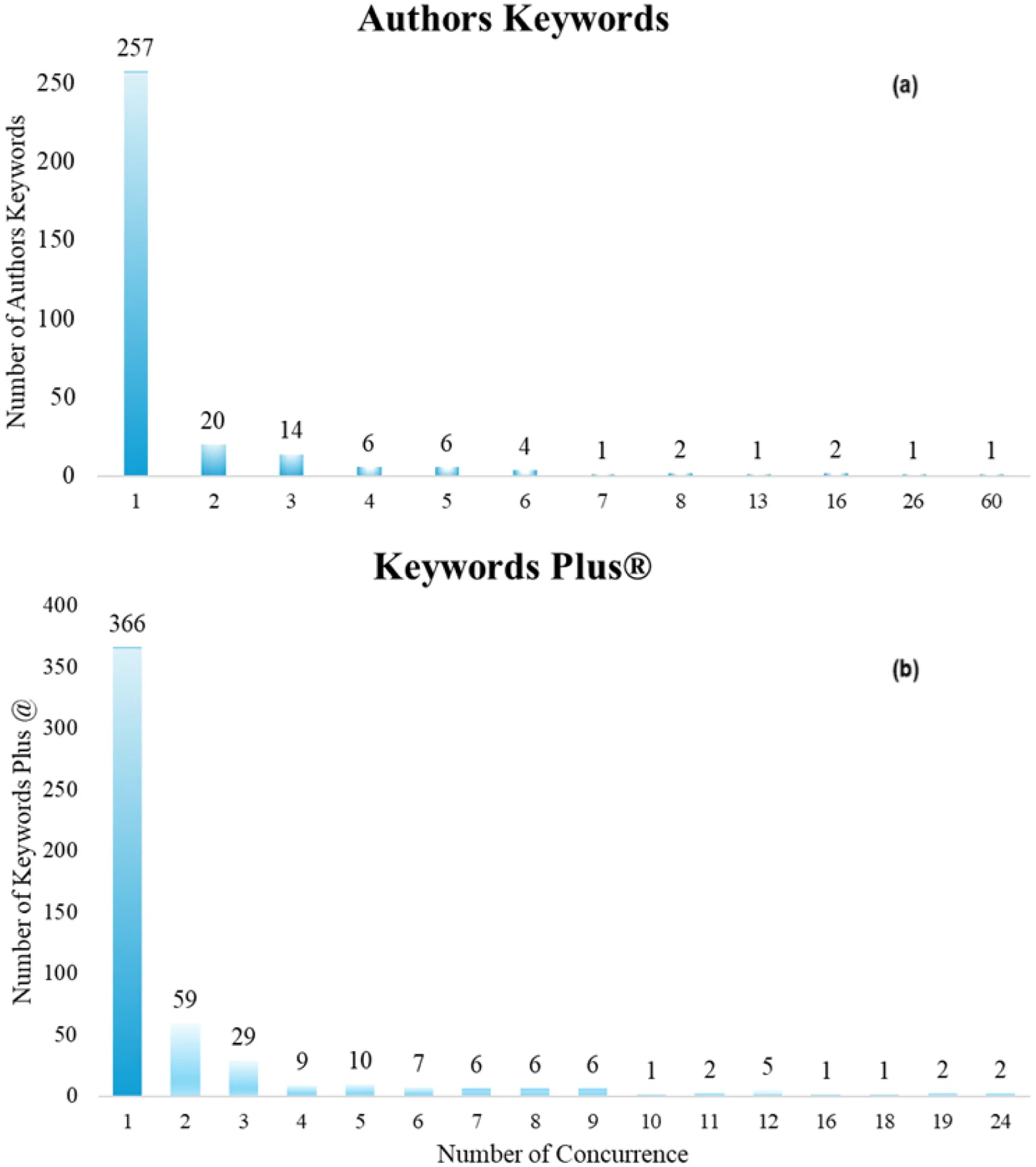
Author's keywords (a) and Keywords Plus® (b) use frequency.

In the 121 papers, authors used a total of 314 author's keywords. These keywords were found to have a frequency of use ranging from 1 to 60 (Figure 5a).

Eighty-two of the keywords (257) were only used on one occasion within the document set. Applying Zipf's law to estimate the number of keywords to be considered as the most frequently used by the co-authors in the document set, it was found that 18 words (the square root of 314) should have the most frequent use. Eighteen were found with a frequency of use of 5 or more, being considered the most frequently used keywords. In addition to *sarcopenia* (60 co-occurrences), *physical activity* (26 occurrences), and *cancer* (16 occurrences), other terms such as *exercise* (16 co-occurrences), *cachexia* (13 co-occurrences), or *body composition* and *nutrition* (both with 8 co-occurrences) stood out. Figure S3 shows the 18 most used keywords and their frequency of use.

**Figure 6.**
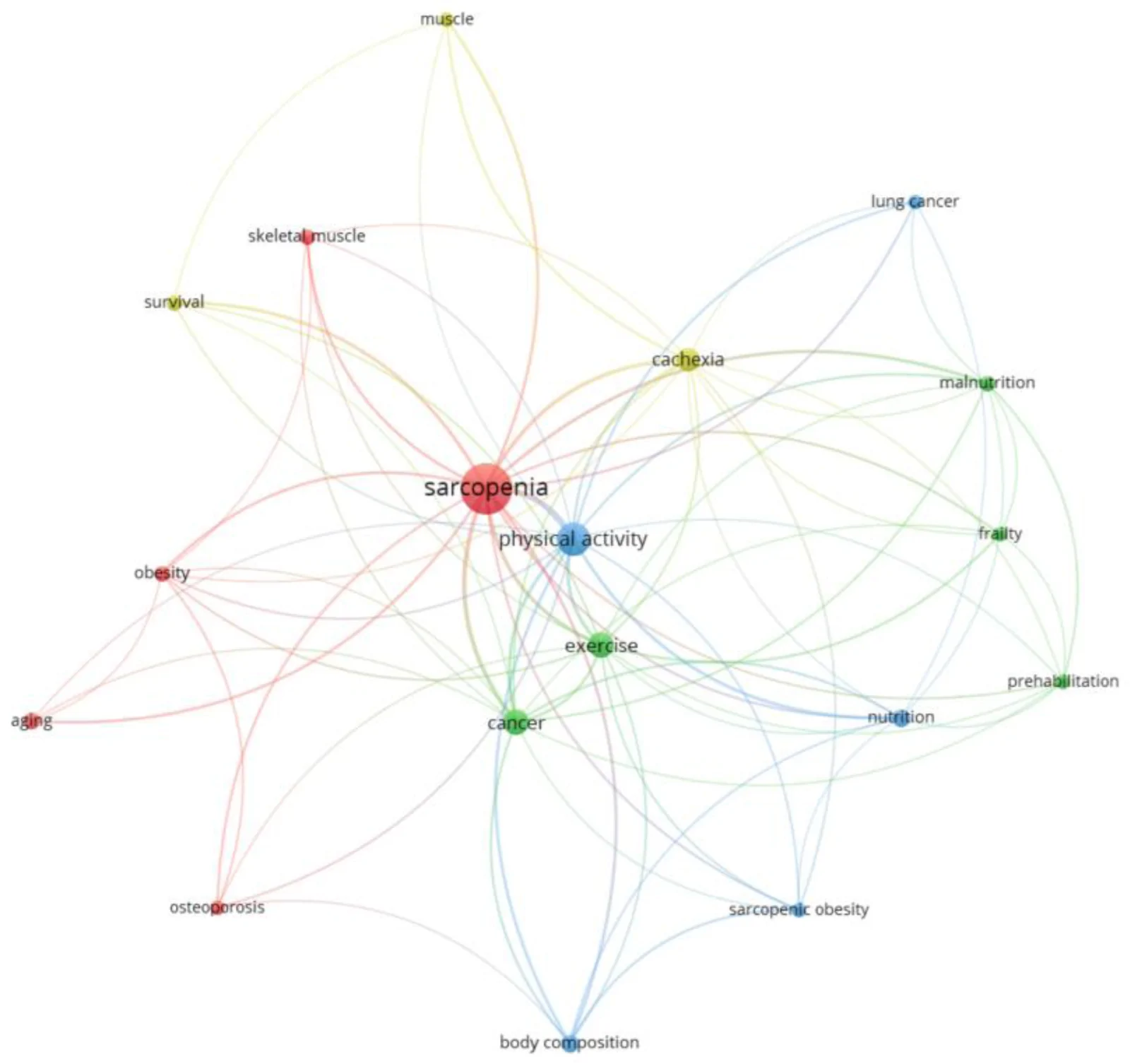
Author keywords co-occurrence graph. Node size: occurrences. Color: cluster.

In the co-occurrence analysis with these keywords, thematic clusters were found (Figure 6). The three most numerous clusters were formed by the terms *sarcopenia*, *obesity*, *osteoporosis*, *aging*, and *skeletal muscle* (Red Cluster), *physical activity*, *nutrition*, *body composition*, *sarcopenic obesity*, and *lung cancer* (Blue Cluster), and *cancer*, *exercise*, *prehabilitation*, *frailty*, and *malnutrition* (Green Cluster). There was also a fourth cluster with terms such as *cachexia*, *survival*, and *muscle* (Yellow Cluster). The terms *cancer*, *skeletal muscle*, *malnutrition*, *nutrition*, and *prehabilitation* had the most recent mean year of publication (Figure S4).

**Figure 7.**
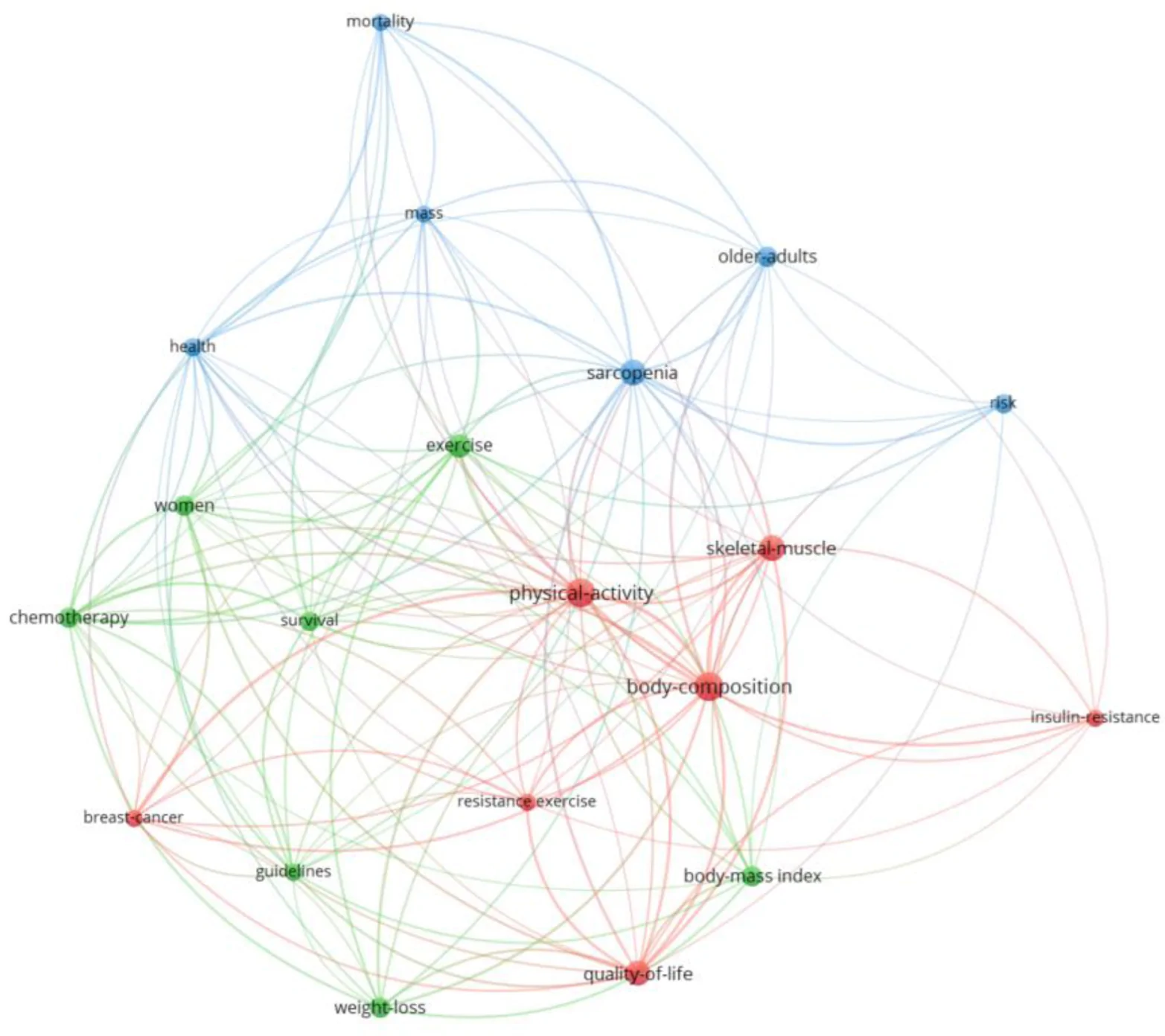
Author keywords graph. Node size: occurrences. Color: cluster.

Finally, 512 Keywords Plus® were found. These had a frequency of occurrence between 1 and 25 (Figure 5b). Applying Zipf's law, it was estimated that the prominent terms should be the 23 with the most co-occurrences. Twenty-six terms were found with 8 or more co-occurrences, while 20 were found with 9 or more, and these were considered to be the most prominent terms. Figure S5 shows these terms and their frequency of occurrence, highlighting terms such as *physical activity* (25 articles), *body composition* (24 documents), or *skeletal muscle* (20 documents). Figure 7 shows the three thematic clusters found after the co-occurrence analysis with the 20 most frequent Keywords Plus®. The first thematic cluster was formed around the three most frequent terms: *physical activity*, *body composition*, and *skeletal muscle*, as well as others such as *quality of life* or *breast cancer* (Red Cluster). Another thematic cluster was formed around terms such as *weight loss*, *exercise*, or *chemotherapy* (Green Cluster). The last cluster was formed around terms such as *sarcopenia*, *older adults*, *mortality*, or *health* (Blue Cluster). Of these terms, those with the most recent year of publication were *survival*, *guidelines*, *older adults*, and *insulin resistance* (Figure S6).

## 4. Discussion

The aim of this study was to evaluate the scientific trends and patterns in the relationship between cancer, sarcopenia, and physical activity. A quantitative bibliometric analysis was conducted, applying traditional laws [34],[35],[39],[54]–[56]. Annual publication trends were analyzed, and WoS categories and journals with the highest number of documents were identified, as well as the most prominent countries in terms of publications and the most productive and prominent authors. Finally, the most cited articles and the most frequently used keywords and Keywords Plus® were presented.

### 4.1. Documents and publication trends

A total of 121 publications were analyzed. The first documents related to this topic were published in 2000 [57],[58]. Although not presenting subject-specific research, they were found to be related to physical activity, sarcopenia, and cancer. The first paper to find a specific relationship between the three objects was published in 2001 [59]. This research was based on a sample of 53 premenopausal women with breast carcinoma; the authors concluded that, given the sarcopenic obesity developed by these women in the absence of physical activity, interventions focused on exercise, especially lower body strength training, were needed to prevent weight gain. During the following years, the object of study entered a phase of research that was a precursor to the exponential growth experienced in recent years. From 2012 to the present, the subject has shown exponential growth with an *R^2^* of 91%, confirming the first of the hypotheses of this study. This finding ensures the existence of an active critical mass that is currently driving this object of study. It is particularly noteworthy that publications have nearly tripled since 2019. Based on these results, it appears that there is a considerable critical mass actively working on this topic, which is consistent with Price's Law [35]. Similar results were observed in previous bibliometric analyses. Notably, the year 2019 marked a significant increase in interest, with an R² value of 70%. However, this study only focused on the topic of sarcopenia and cancer [31] and used a different methodology [34],[35],[39],[54]–[56]. Another bibliometric analysis focusing on breast cancer and physical activity also showed similar exponential growth with publications from 2001 to 2021 [60]. In this sense, our work highlights the relevance of the triad of sarcopenia, PA, and cancer and explores new lines of research as well as emerging authors in this subject.

### 4.2. Categories, journals, authors, and countries

The origin of sarcopenia in cancer patients is multifactorial, involving metabolic, environmental, and inflammatory factors [19] and contributing factors such as malnutrition and physical inactivity [7],[28]. Perhaps because of this, scientific interest in the topic spans various subject areas. Therefore, one of our objectives was to identify the WoS categories with the highest number of publications on this topic.

The two categories with the most publications were *oncology*, which is not surprising, as three prolific and prominent authors publish their work in this category (Newton, R. U. from Australia, Demark-Wahnefried, W. from the USA, and Raynard, B. from France). This category covers research areas related to oncology, health sciences services, rehabilitation, gynecology, and obstetrics. It fits journals such as *Cancers*, which was the journal with the highest number of publication, and contains important scientific contributions such as “cachexia and sarcopenia in older adults with cancer: A comprehensive review” [61], which is a document between the most cited papers, or “Effect of exercise on sarcopenia among cancer survivors: A systematic review” [62]. However, this last paper does not belong to the 36 most cited articles. This review, with publications up to 2021, only contains 7 randomized clinical trials [63]–[69], which shows that, despite being a field of growing scientific interest, much research is still needed to help elucidate the impact of physical activity on cancer-related sarcopenia [7],[62]. This study found promising results, with six of the seven studies showing that exercise increased skeletal muscle by 2.1% to 12.8% compared to control groups; three studies reported sarcopenia reversal in the exercise groups.

In this category, we also find the third most cited article, entitled “Changes in weight, body composition, and factors influencing energy balance among premenopausal breast cancer patients receiving adjuvant chemotherapy”, which was previously mentioned to be the first specific article published. In addition, one of its co-authors was Demark-Wahnefried, W., one of the top authors with the most citations; this paper was published in the *Journal of Clinical Oncology*, one of the leading journals [59].

Other important journals in this category that also belonged to the core journals were the *European Journal of Clinical Nutrition*, *Bulletin du Cancer*, and *Breast Cancer Research and Treatment*. Journals such as the *Journal of Clinical Oncology* or *breast cancer research and treatment* also formed the core journals in a previous biomedical review on breast cancer and physical activity [60]. The second category with the most papers was *dietary nutrition*. The next categories with the most papers covered the areas of *geriatrics, gerontology*, *general internal medicine*, *endocrinology, metabolism*, and *health sciences services*.

Despite being an object of study containing terms such as PA and sarcopenia, there are surprisingly few publications related to categories such as rehabilitation and sport science, with 7 and 6 papers, respectively. This may be because this is a relatively new topic, and these categories are still emerging in a field that is so far focused on other areas.

Although it was possible to establish a core of journals by applying Bradford's law, due to the great peripheral dispersion of journals in terms of their number of publications, it was not possible to segment the journals into the three classic Bradford zones, which can be understood as a relatively new topic in terms of specific research in this field. The journals with the highest number of publications were *Cancers* and *Journal of Cachexia, Sarcopenia and Muscle*, both with 5 papers. These journals were also among the top 10 in the previous bibliometric analysis [31]. However, they have only 1 article among the 36 most cited [61],[70]. In contrast, the journal *Frontiers in Physiology*, which is another of the core journals, presents 3 papers related to this topic. These articles focus on the physiological mechanisms underlying sarcopenia and associated symptoms and on the effects of exercise on muscle tissue; or, in general terms, how physical activity can be a therapeutic prevention [71]–[73]. They are included in the thematic category of physiology, which contains 4 papers among the 36 most cited papers [53],[71]–[73]. In this journal, we also find the American researcher Ferrucci, L., another prominent author with contributions such as “Myosteatosis in the context of skeletal muscle function deficit: An interdisciplinary workshop at the national institute on aging” [71] and who also published in the category *geriatrics and gerontology* (the third category with the highest number of articles) [70]. One other journal with 3 papers between the most cited is the *Current Opinion in Clinical Nutrition and Metabolic Care*, with publications such as “Exercise treatment to counteract protein wasting of chronic diseases” [74], “Omega-3 fatty acids and protein metabolism: enhancement of anabolic interventions for sarcopenia” [75], or “Physical activity and exercise as countermeasures to physical frailty and sarcopenia” [76].

The most cited article, “Lack of exercise is a major cause of chronic diseases”, published in the journal *Comprehensive Physiology* by Booth et al. [53], was also published in physiology categories. However, none of its authors were identified as prolific or prominent authors, and the journal did not belong to the core. In contrast, it was published in 2012, as we found temporal continuity and exponential growth in the topic. This article discusses, in general terms, physical activity as a primary prevention against chronic conditions, including cancers or sarcopenia. The second most cited paper was “Sarcopenia: A time for action. An SCWD position paper” with a total of 409 citations [70]. Not surprisingly, it has 7 prominent authors as co-authors: Muscaritoli, M., Landi, F., Ferrucci, L., Jatoi, A. Laviano, A., and Prado, C. M., researchers from Italy, USA, and Canada. All of them form the collaborative group (cluster green) linked to the red cluster through Laviano, A.

Among the most productive and prominent authors, of the 768 authors, 27 were found to be productive and 22 prominent, confirming the second hypothesis. Newton R. stands out with 2 publications among the most cited [77],[78]. This author belongs to the yellow cluster formed also by another prominent author, Galvao, D. A., who also has 2 papers among the most cited. Both are Australian, one of the 10 countries with the most published papers on this topic (specifically 9, for a total of 799 citations). Among the works of these authors, we find publications like “Changes in muscle, fat and bone mass after 36 weeks of maximal androgen blockade for prostate cancer” [77] and “Exercise in prevention and management of cancer” [78], both among the 36 most cited according to the H-index. This review article highlights the general benefits of regular physical exercise for cancer patients. It shows that exercise can improve surgical outcomes, relieve symptoms, manage side effects of treatments like radiotherapy and chemotherapy, enhance psychological health, maintain physical function, and prevent fat gain and loss of muscle and bone mass, thereby reducing the risk of sarcopenia [78].

Other prominent authors were Landi, F., an Italian researcher within the research group formed by Muscaritoli, M. and Biolo, G.L., also Italian, and by Ferruci, F. Jatoi, A., and Mohile, S. G., American authors with 2 articles among the most cited. Among them, we highlight “Physical activity and exercise as countermeasures to physical frailty and sarcopenia” [76]. This review provides a comprehensive summary of the available evidence in support of physical activity as a remedy for physical frailty and sarcopenia. A critical feature shared by sarcopenia and frailty is the reduction in physical function, which has a negative impact on the quality of life of people with or surviving cancer [13],[79]. Specifically, the United States, led by researchers such as Ferrucci, L. and Jatoi, A., and Italy, led by researchers such as Muscaritoli and M. Landi, F., are the countries with the highest and third highest number of papers published on this topic, respectively. However, despite those being the countries with the most publications, the country with the most international collaborations was Italy. In particular, Italy presents collaborative networks with countries such as Switzerland, Brazil, USA, or Germany, highlighting the author Laviano A. for his collaborative networks with researchers of Canada, Slovenia, Spain, Germany, Australia, Brazil, Switzerland, or USA [70],[80]. In line with our results, a previous bibliometric analysis showed the USA as the most prominent country [60]. However, in other bibliometric analysis about cancer and sarcopenia, the countries with more publications were Japan and China, including researchers such as Maehara, Y. [31].

When analyzing the keywords and keywords added to the documents, a group of terms was found to be used more frequently, which confirms the third hypothesis. The most numerous clusters were related to the question: who or what is affected by sarcopenia? Terms like cancer, aging, muscle, and skeletal muscle were prominent [13],[57],[61],[77]. Other terms related to contributing or secondary factors to sarcopenia related to cancer included osteoporosis, malnutrition, body composition, cachexia, survival, and frailty [53],[61],[76]. Additionally, terms related to therapeutic options for addressing sarcopenia in cancer patients, such as physical activity, nutrition, and exercise, were noted [29],[68],[75],[76],[81].

Similar results were found in a previous systematic review on breast cancer and physical activity, with some of the most frequent keywords being breast cancer, chemotherapy, physical activity, obesity, exercise, and cancer [60]. The recent importance of the term *prehabilitation*, which emphasizes the use of these techniques before surgery to mitigate side effects, was also highlighted. In this regard, a recent review shows the reported benefits of a multimodal treatment based on physical therapy, nutritional support, and psychological support in patients with sarcopenia and colorectal cancer before surgery. Specifically, the benefits include shortened hospitalization, reduced hospital costs, and improved quality of life after surgery in these patients [82]. The recent emergence of this term indicates the current clinical relevance in the approach to people with cancer by introducing a preventive approach that improves the patient's physical function, nutritional status, and psychological well-being prior to treatment.

### 4.3. Practical implications and future lines

The findings of this study are valuable for publishers, junior researchers, and experienced academics. They can use this information to make informed decisions, identify key collaborators and references, and expand their collaborative networks.

In this sense, of the 15 journals that make up the core and the 96 journals in zone 1, there is a great peripheral dispersion; in addition, of these 96 journals, only one article has been published on the topic. Despite being a field of great interest and scientific relevance, we assume that this research topic is still in the process of scientific development, so there are still no specialized journals on the topic. In the same line, out of the 761 authors, only 22 were prominent authors, with between 2 and 1 publications in this field. In addition, we did not observe a large collaborative network between the co-authors. These are important facts, as future research can widely develop this topic.

A future line of research could focus on more experimental studies with longer follow-up periods to determine the optimal exercise type and dose for reversing and/or preventing sarcopenia in patients with various types of cancer [62].

### 4.4. Limitations

This study was based on databases indexed in the WoS core collection, which, despite being one of the most prestigious and widely used databases in the scientific community, introduces a selection bias by not including research from non-indexed journals. This choice was made to guarantee the quality of the documents analyzed, ensuring that they were all peer-reviewed and met the strict WoS indexing criteria. However, this choice also implied some limitations by not including some high-impact research that is published in journals indexed in other databases. In this sense, grey literature, such as conference proceedings, technical reports, etc., was also not included. However, including this type of literature would have prevented ensuring peer review of the documents, traceability of sources, and availability of data, among other limitations. Including other terms would have generated new selection biases, including documents with concepts that are not synonymous. In relation to this choice bias, some regions or countries may have been underrepresented. Researchers from some Asian countries, some Eastern European countries, or other emerging economies might be publishing in national or local journals not indexed in WoS, therefore not being represented in our research. Another limitation may have been related to the exclusion of animal studies, as preclinical studies could complement our research by providing a basis for understanding the underlying biological mechanisms related to sarcopenia, physical activity, and cancer. The search vector also limited access to papers related to the field of study. The aim of the study was to analyze research published in journals indexed in WoS and related to sarcopenia, physical activity, and cancer. In trying to be conceptually precise, we could not include other terms related to physical activity, such as exercise, training, and sports, among others.

## 5. Conclusion

Research on the relationship between cancer, sarcopenia, and physical activity is in an exponential growing phase. This object of study has been approached from numerous thematic areas, with articles related to up to 36 WoS thematic categories. Among the 761 authors, Newton, R. U., Galvao, D. A., Muscaritoli, M., Landi, F., Ferrucci, L., Jatoi, A., Biolo, G., Winett, R. A., and Mohile, S. G. stood out as the most prolific and prominent authors, with 2 papers between the 36 most cited.

Thirty-six papers with 37 or more citations were identified as the most cited papers in the topic. Countries such as the USA and Italy contributed the greater number of documents, and the journals *Cancers* and *Journal of Cachexia, Sarcopenia and Muscle* were the ones with the most publications. Regarding keywords, a differentiation was found between author keywords and Keywords Plus®. Both identified various thematic clusters related to words such as cancer, physical activity, aging, muscle, skeletal muscle, osteoporosis, malnutrition, body composition, cachexia, survival, and frailty.

Current trends reveal that there is a large critical mass that continues to contribute to a moderate volume of annual publications, motivated both by research areas that have evolved over time and new areas of interest, such as the effects of prehabilitation.

## Use of AI tools declaration

The authors declare they have not used Artificial Intelligence (AI) tools in the creation of this article.


