# Supplementary material for: Overview and current status of published research on cancer, sarcopenia and physical activity: A bibliometric analysis
Source: AIMS Public Health. 2025 Jun 23;12(3):632–56. doi: 10.3934/publichealth.2025033 (PMC12538244; doi:10.3934/publichealth.2025033)
Supplement: Supplementary file 1 [file publichealth-12-03-033-s001.pdf]

Public Health

AIMS Public Health, 12(3): 632–656.
DOI: 10.3934/publichealth.2025033
Received: 05 February 2025
Revised: 28 March 2025
Accepted: 24 April 2025
Published: 23 June 2025

https://www.aimspress.com/journal/aimsph

Review

Overview and current status of published research on cancer, sarcopenia and physical activity: A bibliometric analysis

J. A. Parraca[1,2], D. Salas-Gómez[1,*], R. Dinis[3,2], A. Denche-Zamorano[4,*], A. Vega-Muñoz[5,6] and P. Tomas-Carus[1,2]

[1] Departamento de Desporto e Saúde, Escola de Saúde e Desenvolvimento Humano, Universidade de Évora, 7004-516 Evora, Portugal
[2] Comprehensive Health Research Centre (CHRC), University of Evora, 7004-516 Evora, Portugal
[3] Medical Oncology, Hospital do Espirito Santo de Evora EPE, Evora, Portugal
[4] Promoting a Healthy Society Research Group (PHeSO), Faculty of Sport Sciences, University of Extremadura, 10003 Caceres, Spain
[5] Centro de Investigación en Educación de Calidad para la Equidad, Universidad Central de Chile, Santiago 8330601, Chile.
[6] Facultad de Ciencias Empresariales, Universidad Arturo Prat, Iquique 1110939, Chile.

* **Correspondence:** Email: D. Salas-Gómez: diana.salas.gom@gmail.com; A. Denche-Zamorano: denchezamorano@unex.es.

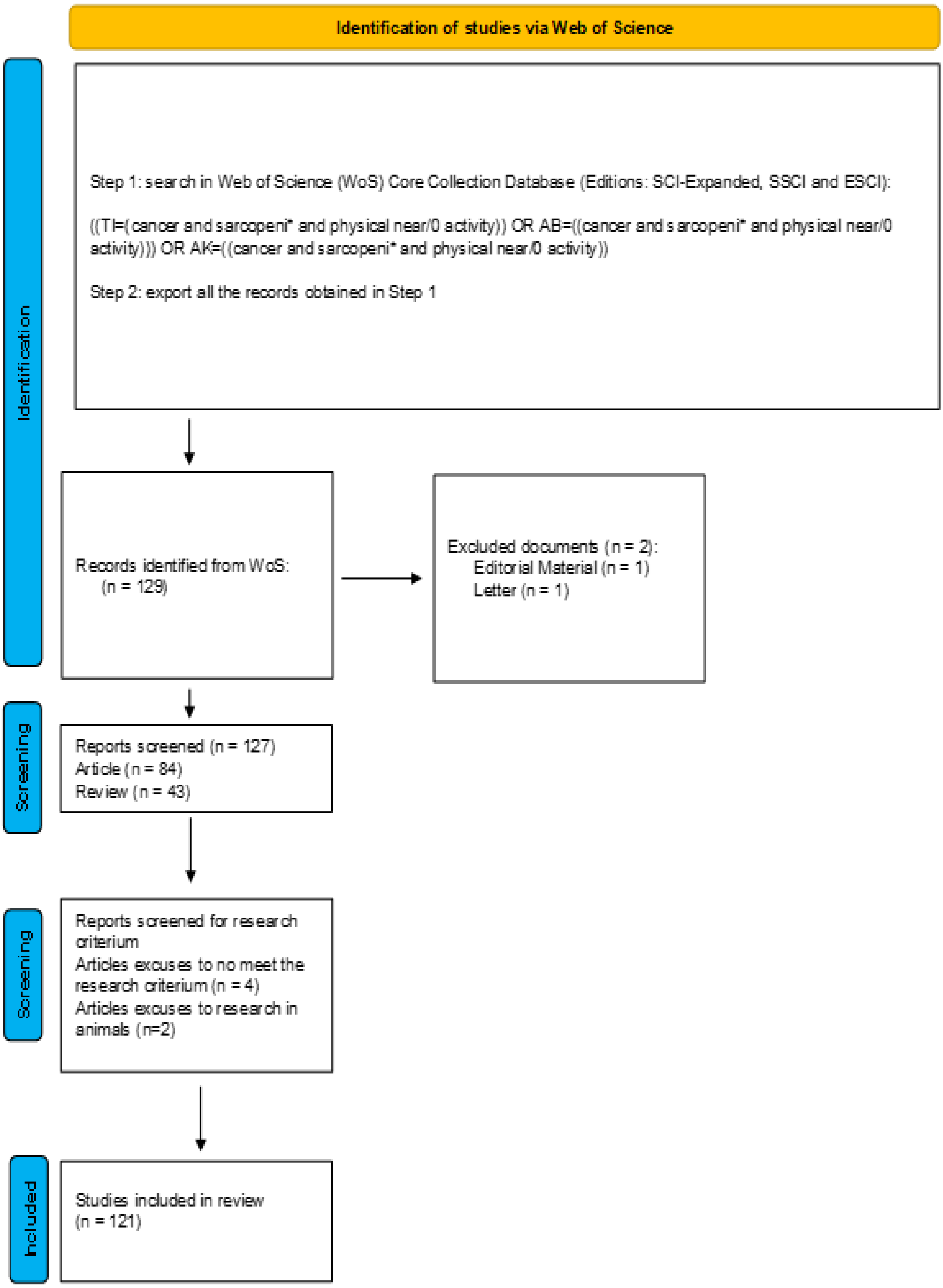

Figure S1. Flowchart.

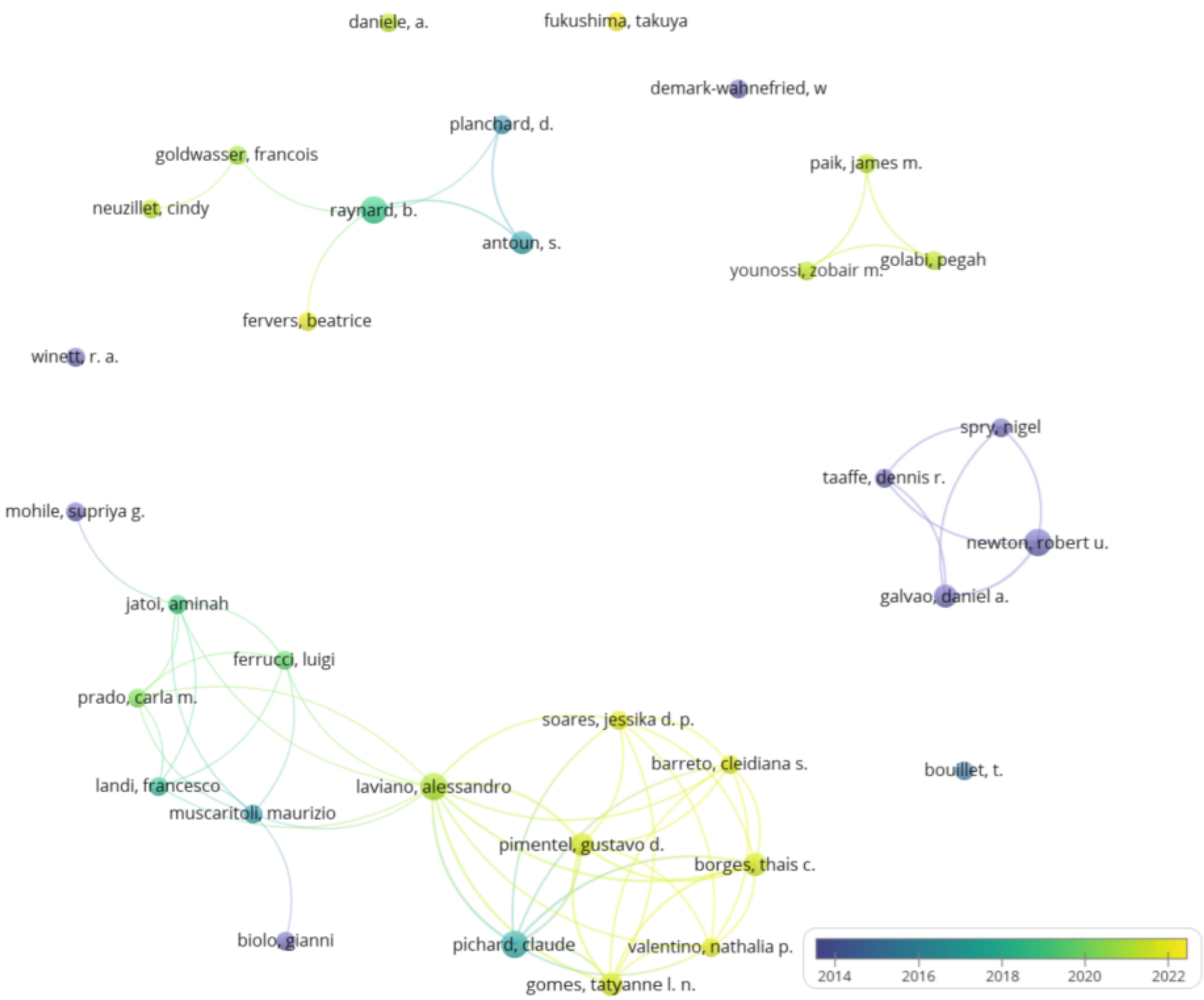

Figure S2. The co-authors according to the average year of their publications.

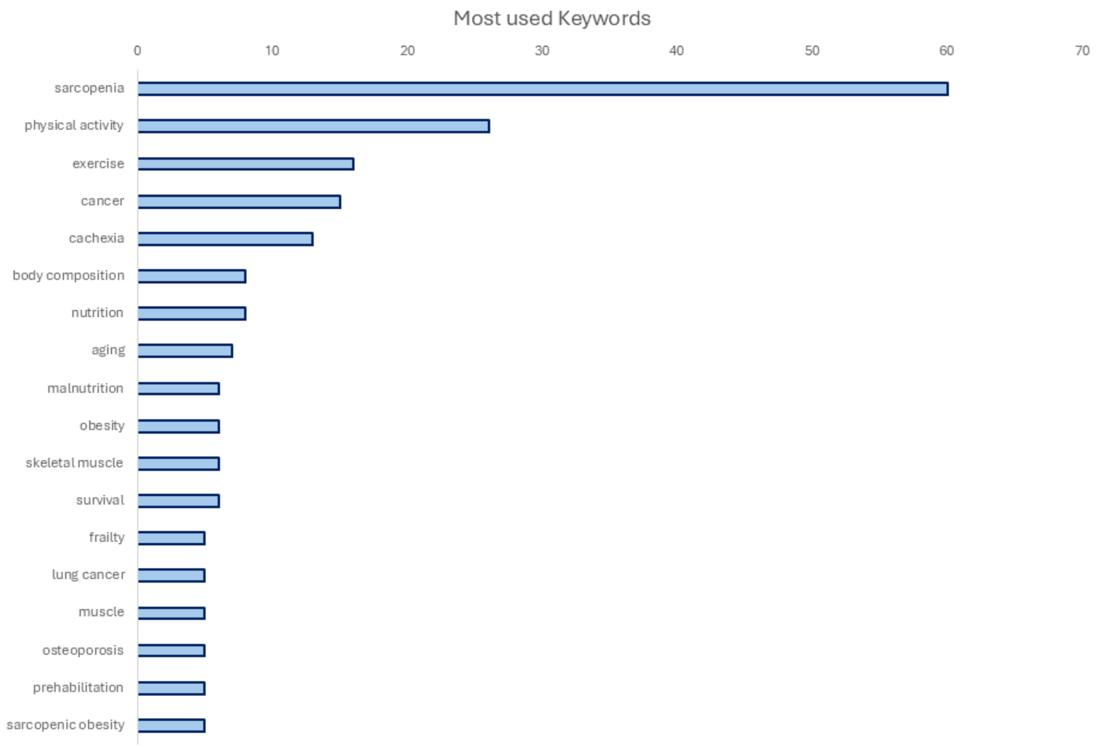

Figure S3. 18 most used keywords.

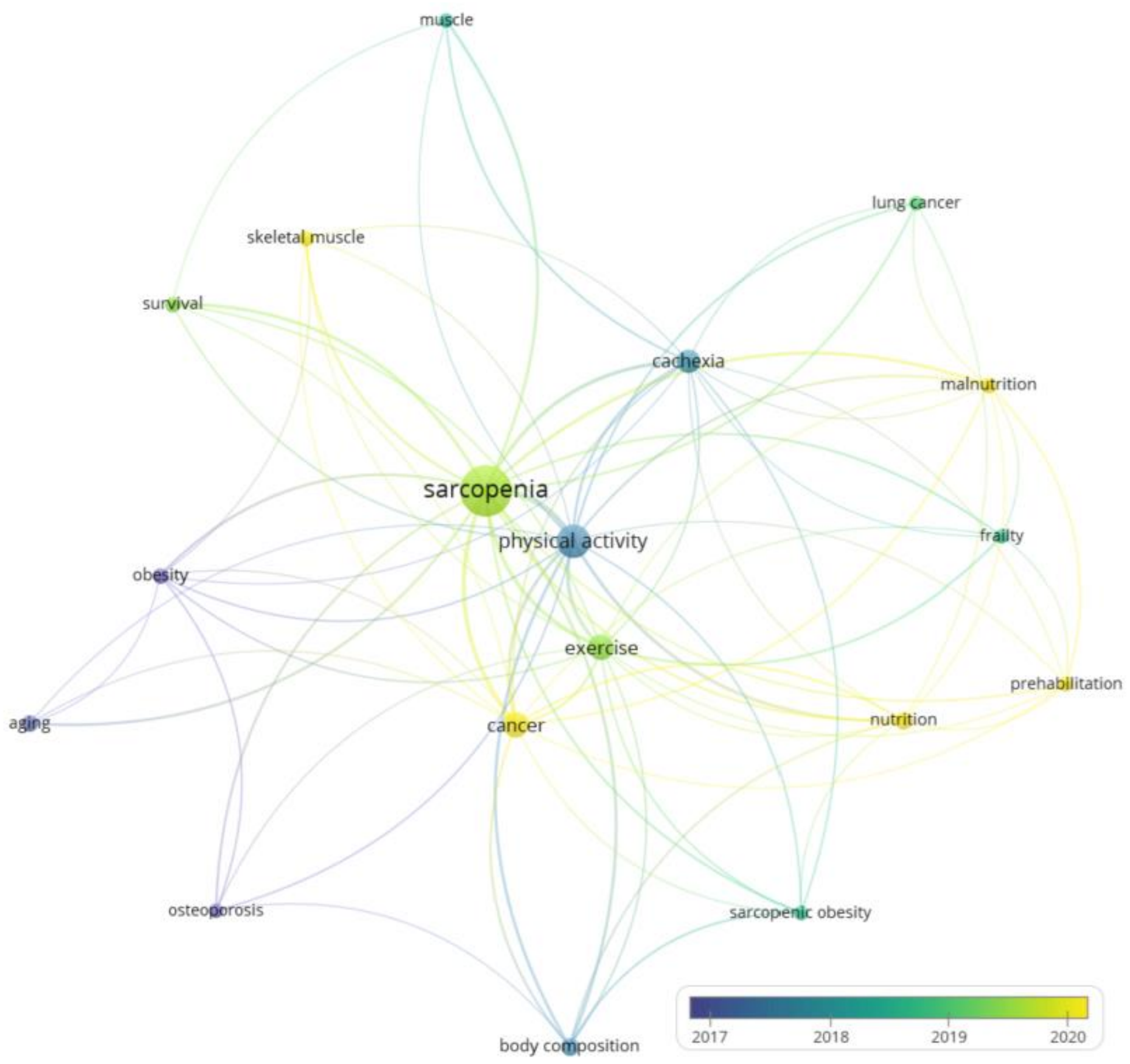

Figure S4. AK as 5_0 Avg Pub Years.

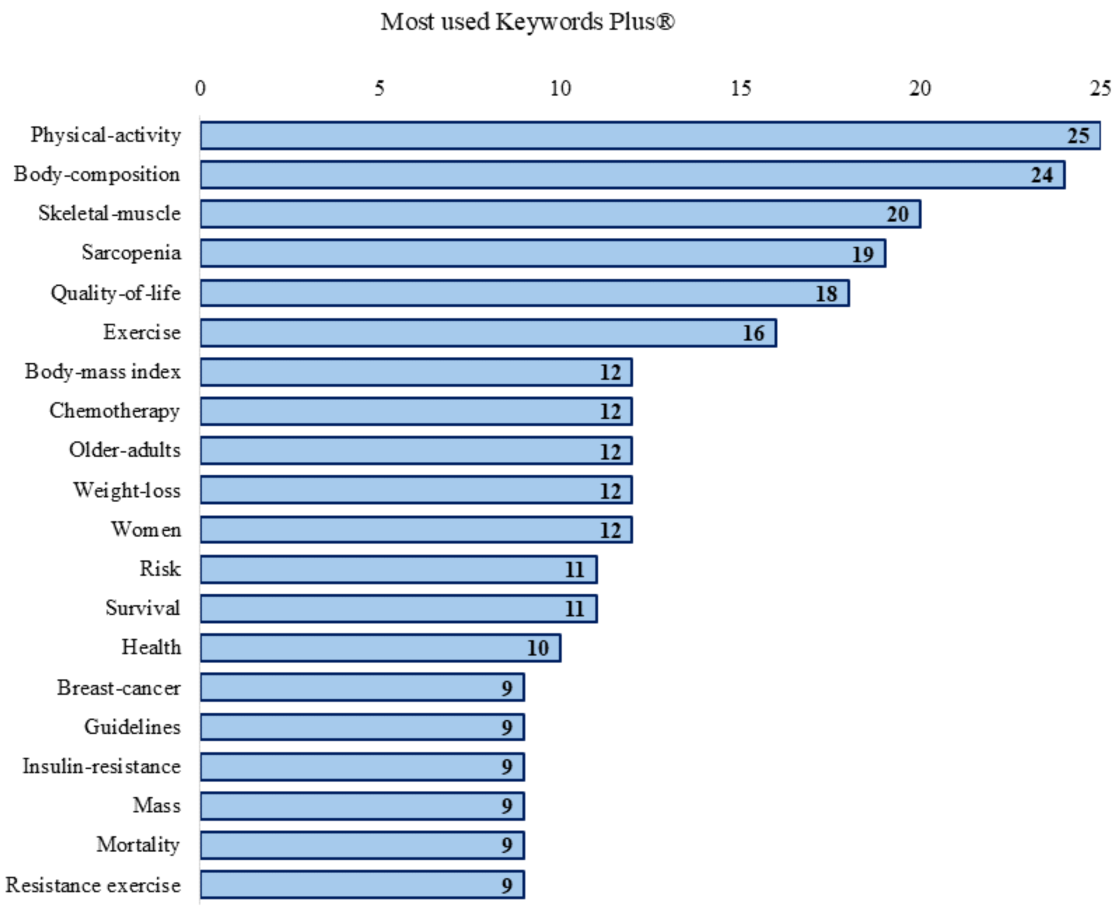

Figure S5. Keyword Plus and their frequency of occurrence.

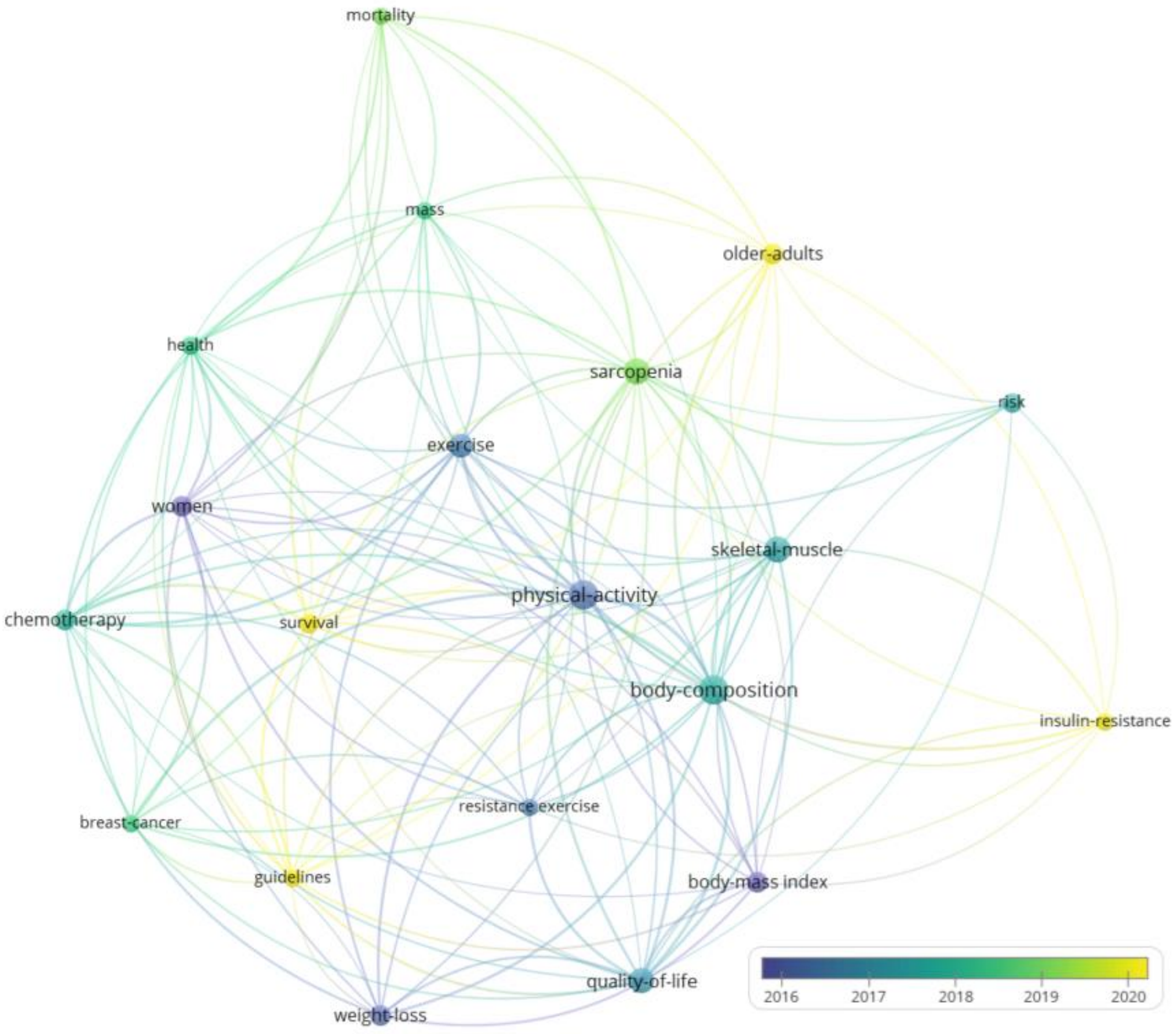

Figure S6. KP AS 5_0 Avg Pub Years.

Table S1. Most cited papers.

Nomber	Author Full Names	Title	Publisher	Document Type	Author Keywords	Keywords Plus	Times Cited, WoS Core	Publication Year	WoS Categories	Web of Science Index	Pubmed Id	Open Access Designations	UT (Unique WOS ID)
J	Booth, FW; Roberts, CK; Laye, MJ	Lack of Exercise Is a Major Cause of Chronic Diseases	COMPREHENSIVE PHYSIOLOGY	English	Article		BODY-MASS INDEX; ALL-CAUSE MORTALITY; TIME PHYSICAL-ACTIVITY; GROWTH-FACTOR-I; NEGATIVE-ENERGY BALANCE; LIFE-STYLE INTERVENTION; MODIFIABLE RISK-FACTORS; HUMAN SKELETAL-MUSCLE; FATTY LIVER-DISEASE; NUTRITION EXAMINATION SURVEY	1515	2012	Physiology	Science Citation Index Expanded (SCI-EXPANDED)	Green Accepted	WOS:000314650800009
J	Bauer, J; Morley, JE; Schols, AMWJ; Ferrucci, L; Cruz-Jentoft, AJ; Dent, E; Baracos, VE; Crawford, JA; Doehner, W; Heymsfield, SB; Jatoi, A; Kalantar-Zadeh, K; Lainscak, M; Landi, F; Laviano, A; Mancuso, M; Muscaritoli, M; Prado, CM; Strasser, F; von Haehling, S; Coats, AJS; Anker, SD	Sarcopenia: A Time for Action. An SCWD Position Paper	JOURNAL OF CACHEXIA SARCOPENIA AND MUSCLE	English	Article	Sarcopenia; Cachexia; Geriatric assessment; Muscle; Skeletal; Muscle strength	SKELETAL-MUSCLE MASS; OLDER-ADULTS; INTERNATIONAL-CONFERENCE; CLINICAL-PRACTICE; CACHEXIA SYNDROME; PHYSICAL FUNCTION; DOUBLE-BLIND; VITAMIN-D; CANCER; ULTRASOUND	409	2019	Geriatrics & Gerontology; Medicine, General & Internal	Science Citation Index Expanded (SCI-EXPANDED); Social Science Citation Index (SSCI)	Green Published, gold	WOS:000490285400001
J	Demark-Wahnefried, W; Peterson, BL; Winer, EP; Marks, L; Aziz, N; Marcom, PK; Blackwell, K; Rimer, BK	Changes in weight, body composition, and factors influencing energy balance among premenopausal breast cancer patients receiving adjuvant chemotherapy	JOURNAL OF CLINICAL ONCOLOGY	English	Article		HEALTH TRIAL FEASIBILITY; QUALITY-OF-LIFE; MINORITY POPULATIONS; MEGESTROL-ACETATE; GAIN; WOMEN; DIAGNOSIS; EXERCISE; THERAPY; REHABILITATION	389	2001	Oncology	Science Citation Index Expanded (SCI-EXPANDED)		WOS:000168484000003
J	Batsis, JA; Mackenzie, TA; Barre, LK; Lopez-Jimenez, F; Bartels, SJ	Sarcopenia, sarcopenic obesity and mortality in older adults: results from the National Health and Nutrition Examination Survey III	EUROPEAN JOURNAL OF CLINICAL NUTRITION	English	Article		BODY-MASS INDEX; SKELETAL-MUSCLE MASS; BIOELECTRICAL-IMPEDANCE; PHYSICAL-DISABILITY; METABOLIC SYNDROME; GRIP STRENGTH; MEN; PREVALENCE; WOMEN; AGE	329	2014	Nutrition & Dietetics	Science Citation Index Expanded (SCI-EXPANDED); Social Science Citation Index (SSCI)	Bronze	WOS:000341168600005
J	Romanello, V; Sandri, M	Mitochondrial Quality Control and Muscle Mass Maintenance	FRONTIERS IN PHYSIOLOGY	English	Review	atrophy; mitochondria; fission; fusion; biogenesis; autophagy; muscle; sarcopenia	UBIQUITIN-PROTEASOME SYSTEM; HUMAN SKELETAL-MUSCLE; TARGETED ANTIOXIDANTS PROTECT; AGE-RELATED-CHANGES; OXIDATIVE STRESS; PHYSICAL-ACTIVITY; PERMEABILITY TRANSITION; INDUCED PHOSPHORYLATION; STIMULATES AUTOPHAGY; CALORIE RESTRICTION	304	2016	Physiology	Science Citation Index Expanded (SCI-EXPANDED)	Green Published, gold	WOS:000367954900003

J	Biolo, G; Cederholm, T; Muscaritoli, M	Muscle contractile and metabolic dysfunction is a common feature of sarcopenia of aging and chronic diseases: From sarcopenic obesity to cachexia	CLINICAL NUTRITION	English	Article	Sarcopenia; Cachexia; Sarcopenic obesity; Muscle metabolic dysfunction; Pre-cachexia; Muscle contractile dysfunction	RESTING ENERGY-EXPENDITURE; BODY-MASS INDEX; CHRONIC HEART-FAILURE; SKELETAL-MUSCLE; BED-REST; INSULIN-RESISTANCE; CANCER-PATIENTS; PROTEIN-METABOLISM; AMINO-ACID; NEOADJUVANT CHEMOTHERAPY	280	2014	Nutrition & Dietetics	Science Citation Index Expanded (SCI-EXPANDED)		WOS:000347745700001
J	Marzetti, E; Calvani, R; Tosato, M; Cesari, M; Di Bari, M; Cherubini, A; Broccatelli, M; Savera, G; D'Elia, M; Pahor, M; Bernabei, R; Landi, F	Physical activity and exercise as countermeasures to physical frailty and sarcopenia	AGING CLINICAL AND EXPERIMENTAL RESEARCH	English	Review	Skeletal muscle; Physical performance; Exercise; Resistance training; Endurance training	LIFE-STYLE INTERVENTIONS; BODY-COMPOSITION; OLDER-ADULTS; MUSCLE FUNCTION; INDEPENDENCE; PERFORMANCE; ELDERS; PREVALENCE; MECHANISMS; DISABILITY	225	2017	Geriatrics & Gerontology	Science Citation Index Expanded (SCI-EXPANDED); Social Science Citation Index (SSCI)		WOS:000396128000006
J	Castillo, EM; Goodman-Gruen, D; Kritz-Silverstein, D; Morton, DJ; Wingard, DL; Barrett-Connor, E	Sarcopenia in elderly men and women - The Rancho Bernardo Study	AMERICAN JOURNAL OF PREVENTIVE MEDICINE	English	Article		BIOELECTRICAL-IMPEDANCE ANALYSIS; BODY-COMPOSITION; MUSCLE MASS; FAT-FREE; OLDER; AGE; EPIDEMIOLOGY; RISK	222	2003	Public, Environmental & Occupational Health; Medicine, General & Internal	Science Citation Index Expanded (SCI-EXPANDED)		WOS:000185720200007
J	Correa-de-Araujo, R; Addison, O; Miljkovic, I; Goodpaster, BH; Bergman, BC; Clark, RV; Elena, JW; Esser, KA; Ferrucci, L; Harris-Love, MO; Kritchevsky, SB; Lorbergs, A; Shepherd, JA; Shulman, GI; Rosen, CJ	Myosteatosis in the Context of Skeletal Muscle Function Deficit: An Interdisciplinary Workshop at the National Institute on Aging	FRONTIERS IN PHYSIOLOGY	English	Review	myosteatosis; intermuscular adipose tissue; intramuscular adipose tissue; intramyocellular lipids; skeletal muscle function deficit; muscle quality; aging; mobility-disability	INTRAMUSCULAR ADIPOSE-TISSUE; CREATINE METHYL-D(3) DILUTION; GAIT-SPEED DECLINE; INSULIN-RESISTANCE; FAT INFILTRATION; OLDER-ADULTS; COMPUTED-TOMOGRAPHY; BODY-COMPOSITION; TRUNK MUSCLE; LONGITUDINAL CHANGES	219	2020	Physiology	Science Citation Index Expanded (SCI-EXPANDED); Social Science Citation Index (SSCI)	Green Published, gold	WOS:000563217800001
J	Galvao, DA; Spry, NA; Taaffe, DR; Newton, RU; Stanley, J; Shannon, T; Rowling, C; Prince, R	Changes in muscle, fat and bone mass after 36 weeks of maximal androgen blockade for prostate cancer	BJU INTERNATIONAL	English	Article	lean mass; fat mass; bone mass; androgen deprivation therapy	X-RAY ABSORPTIOMETRY; DEPRIVATION THERAPY; MINERAL DENSITY; SKELETAL-MUSCLE; HORMONE AGONISTS; BODY-COMPOSITION; SOFT-TISSUE; MEN; OSTEOPOROSIS; CARCINOMA	204	2008	Urology & Nephrology	Science Citation Index Expanded (SCI-EXPANDED)		WOS:000256491600011
J	Anandavadivelan, P; Lagergren, P	Cachexia in patients with oesophageal cancer	NATURE REVIEWS CLINICAL ONCOLOGY	English	Review		QUALITY-OF-LIFE; BODY-MASS INDEX; C-REACTIVE PROTEIN; EORTC QUESTIONNAIRE MODULE; GLASGOW PROGNOSTIC SCORE; NECROSIS-FACTOR-ALPHA; WEIGHT-LOSS; NEOADJUVANT CHEMOTHERAPY; SYSTEMIC INFLAMMATION; NUTRITIONAL-STATUS	180	2016	Oncology	Science Citation Index Expanded (SCI-EXPANDED)		WOS:000371445700007
J	Suzuki, K	Chronic Inflammation as an Immunological Abnormality and Effectiveness of Exercise	BIOMOLECULES	English	Article	cytokine; neutrophil; macrophage; lipopolysaccharides (LPS); free fatty acids (FFA); Toll-like receptor (TLR);	ATTENUATES OXIDATIVE STRESS; GREEN TEA EXTRACT; ENDURANCE EXERCISE; MUSCLE DAMAGE; IMMUNOENDOCRINE	166	2019	Biochemistry & Molecular Biology	Science Citation Index Expanded (SCI-EXPANDED)	Green Submitted, Green Published, gold	WOS:000475301500019

						reactive oxygen species (ROS); anti-inflammatory effect of exercise; aging; non-communicable disease (NCD)	RESPONSES; NEUTROPHIL ACTIVATION; EXHAUSTIVE EXERCISE; VOLUNTARY EXERCISE; MODERATE-INTENSITY; SKELETAL-MUSCLE						
J	Winett, RA; Carpinelli, RN	Potential health-related benefits of resistance training	PREVENTIVE MEDICINE	English	Review	resistance training; strength; musculoskeletal system; lower-back pain; bone mineral density; disease prevention; exercise guidelines	BODY-MASS INDEX; CHRONIC RESISTIVE EXERCISE; VISCERAL ADIPOSE-TISSUE; RESTING BLOOD-PRESSURE; BONE-MINERAL DENSITY; METABOLIC-RATE; RISK-FACTORS; COLORECTAL-CANCER; PHYSICAL-ACTIVITY; MUSCLE STRENGTH	164	2001	Public, Environmental & Occupational Health; Medicine, General & Internal	Science Citation Index Expanded (SCI-EXPANDED); Social Science Citation Index (SSCI)		WOS:000172078500021
J	Evans, WJ	Protein nutrition, exercise and aging	JOURNAL OF THE AMERICAN COLLEGE OF NUTRITION	English	Article; Proceedings Paper	aging; elderly; sarcopenia; muscle mass; diet; protein; strength conditioning	RANDOMIZED CONTROLLED-TRIAL; HUMAN SKELETAL-MUSCLE; RESISTANCE EXERCISE; OLDER MEN; ECCENTRIC EXERCISE; INSULIN-SECRETION; BODY-COMPOSITION; ELDERLY-MEN; ENERGY-REQUIREMENTS; LEUCINE METABOLISM	152	2004	Nutrition & Dietetics	Science Citation Index Expanded (SCI-EXPANDED); Conference Proceedings Citation Index - Science (CPCI-S)		WOS:000226526400004
S	Anderson, LJ; Liu, HM; Garcia, JM	Sex Differences in Muscle Wasting	SEX AND GENDER FACTORS AFFECTING METABOLIC HOMEOSTASIS, DIABETES AND OBESITY	English	Article; Book Chapter		CHRONIC HEART-FAILURE; OBSTRUCTIVE PULMONARY-DISEASE; FREE TESTOSTERONE LEVELS; LEAN BODY-MASS; HORMONE REPLACEMENT THERAPY; ANDROGEN RECEPTOR MODULATOR; CELL LUNG-CANCER; QUALITY-OF-LIFE; INDEPENDENT PROGNOSTIC-FACTOR; ORAL NUTRITIONAL SUPPLEMENT	148	2017	Biochemical Research Methods; Biochemistry & Molecular Biology; Endocrinology & Metabolism; Medicine, Research & Experimental	Book Citation Index – Science (BKCI-S); Science Citation Index Expanded (SCI-EXPANDED)		WOS:000715520200012
J	Dunne, RF; Loh, KP; Williams, GR; Jatoi, A; Mustian, KM; Mohile, SG	Cachexia and Sarcopenia in Older Adults with Cancer: A Comprehensive Review	CANCERS	English	Review	cachexia; sarcopenia; geriatric oncology; geriatric assessment; weight loss; muscle; wasting	RANDOMIZED CONTROLLED-TRIAL; CELL LUNG-CANCER; III CLINICAL-TRIAL; SKELETAL-MUSCLE; PHYSICAL PERFORMANCE; GERIATRIC ASSESSMENT; COMPUTED-TOMOGRAPHY; AEROBIC EXERCISE; GASTRIC-CANCER; DOUBLE-BLIND	128	2019	Oncology	Science Citation Index Expanded (SCI-EXPANDED); Social Science Citation Index (SSCI)	gold, Green Published	WOS:000507382100040
J	Newton, RU; Galvao, DA	Exercise in Prevention and Management of Cancer	CURRENT TREATMENT OPTIONS IN ONCOLOGY	English	Article		ANDROGEN DEPRIVATION THERAPY; RECREATIONAL PHYSICAL-ACTIVITY; QUALITY-OF-LIFE; PROSTATE-CANCER; COLORECTAL-CANCER; PUBLIC-HEALTH; AMERICAN-COLLEGE; CONTROLLED-TRIAL; SPORTS-MEDICINE; RISK	99	2008	Oncology	Science Citation Index Expanded (SCI-EXPANDED)		WOS:000263030300005
J	Hilmi, M; Jouinot, A; Burns, R; Pigneur, F; Mounier, R; Gondin, J;	Body composition and sarcopenia: The next-generation of personalized	PHARMACOLOGY & THERAPEUTICS	English	Review	Cachexia; Chemotherapy; Sarcopenic obesity; Targeted therapy; Toxicity	CELL LUNG-CANCER; SKELETAL-MUSCLE MASS; INDEPENDENT PROGNOSTIC-FACTOR; DOSE-LIMITING	98	2019	Pharmacology & Pharmacy	Science Citation Index Expanded (SCI-EXPANDED); Social Science	Green Submitted, Bronze	WOS:000465052900009

	Neuzillet, C; Goldwasser, F	oncology and pharmacology?					TOXICITY; NEUROMUSCULAR ELECTRICAL-STIMULATION; CREATININE/CYSTATIN C RATIO; GLOMERULAR-FILTRATION-RATE; OBESE ADULT PATIENTS; LONG-TERM OUTCOMES; NEOADJUVANT CHEMOTHERAPY				Citation Index (SSCI)		
J	Bylow, K; Mohile, SG; Stadler, WM; Dale, W	Does androgen-deprivation therapy accelerate the development of frailty in older men with prostate cancer? - A conceptual review	CANCER	English	Review	prostate cancer; androgen-deprivation therapy; frailty; older adults	QUALITY-OF-LIFE; LEAN BODY-MASS; C-REACTIVE PROTEIN; ELDERLY PERSONS; PHYSICAL PERFORMANCE; RADIATION-THERAPY; MUSCLE STRENGTH; ONCOLOGY GROUP; RADICAL PROSTATECTOMY; CARDIOVASCULAR HEALTH	94	2007	Oncology	Science Citation Index Expanded (SCI-EXPANDED)	Bronze	WOS:000251573600003
J	Zinna, EM; Yarasheski, KE	Exercise treatment to counteract protein wasting of chronic diseases	CURRENT OPINION IN CLINICAL NUTRITION AND METABOLIC CARE	English	Article	cachexia; hypertrophy; physical activity; skeletal muscle amino acid metabolism	RESISTANCE EXERCISE; BREAST-CANCER; MUSCLE; WOMEN; CHEMOTHERAPY; INDIVIDUALS; PREVENTION; STRENGTH; QUALITY; MASS	81	2003	Endocrinology & Metabolism; Nutrition & Dietetics	Science Citation Index Expanded (SCI-EXPANDED)		WOS:000180517800013
J	Di Girolamo, FG; Situlin, R; Mazzucco, S; Valentini, R; Toigo, G; Biolo, G	Omega-3 fatty acids and protein metabolism: enhancement of anabolic interventions for sarcopenia	CURRENT OPINION IN CLINICAL NUTRITION AND METABOLIC CARE	English	Review	cachexia; omega-3 fatty acids; physical inactivity; protein metabolism; sarcopenia	POLYUNSATURATED FATTY-ACIDS; FISH-OIL; CANCER; CACHEXIA; WOMEN; SUPPLEMENTATION; INACTIVITY; DIAGNOSIS; OMEGA-3; YOUNG	77	2014	Endocrinology & Metabolism; Nutrition & Dietetics	Science Citation Index Expanded (SCI-EXPANDED)		WOS:000333450400006
J	Keevil, VL; Luben, R; Dalzell, N; Hayat, S; Sayer, AA; Wareham, NJ; Khaw, KT	CROSS-SECTIONAL ASSOCIATIONS BETWEEN DIFFERENT MEASURES OF OBESITY AND MUSCLE STRENGTH IN MEN AND WOMEN IN A BRITISH COHORT STUDY	JOURNAL OF NUTRITION HEALTH & AGING	English	Article	Grip strength; central obesity; sarcopenia; aging	PHYSICAL-ACTIVITY; GRIP STRENGTH; OLDER-ADULTS; BODY-COMPOSITION; HEALTH; SARCOPENIA; MORTALITY; MASS; QUESTIONNAIRE; DEFINITION	72	2015	Geriatrics & Gerontology; Nutrition & Dietetics	Science Citation Index Expanded (SCI-EXPANDED); Social Science Citation Index (SSCI)	Green Accepted, hybrid	WOS:000348024800001
J	Melzer, K; Kayser, B; Pichard, C	Physical activity: the health benefits outweigh the risks	CURRENT OPINION IN CLINICAL NUTRITION AND METABOLIC CARE	English	Article	cardiac arrest; cardiovascular disease; cognitive disorders; diabetes; dehydration; disease; exercise; health; injuries; obesity; osteoporosis; oxidative stress; physical activity; risk; sarcopenia	SKELETAL-MUSCLE; SUDDEN-DEATH; WEIGHT-LOSS; FAT MASS; EXERCISE; WALKING; BODY; METABOLISM; DISEASE; MEN	72	2004	Endocrinology & Metabolism; Nutrition & Dietetics	Science Citation Index Expanded (SCI-EXPANDED)		WOS:000225551400008
J	Trovato, E; Di Felice, V; Barone, R	Extracellular Vesicles: Delivery Vehicles of Myokines	FRONTIERS IN PHYSIOLOGY	English	Review	exercise; muscle cells; exocytosis; extracellular bodies; exosomes	EXOSOME-MEDIATED DELIVERY; HUMAN SKELETAL-MUSCLE; NEUROTROPHIC FACTOR; PHYSICAL-ACTIVITY; MESSENGER-RNA; ENGINEERED EXOSOME; INSULIN-RESISTANCE; ENDURANCE EXERCISE; AEROBIC EXERCISE; CELL	70	2019	Physiology	Science Citation Index Expanded (SCI-EXPANDED)	Green Published, gold	WOS:000467355900001

J	Golabi, P; Gerber, L; Paik, JM; Deshpande, R; de Avila, L; Younossi, ZM	Contribution of sarcopenia and physical inactivity to mortality in people with non-alcoholic fatty liver disease	JHEP REPORTS	English	Article	Sarcopenia; Non-alcoholic fatty liver disease; Physical activity	INSULIN-RESISTANCE; NATIONAL-HEALTH; PREVALENCE; OBESITY; ADULTS; ASSOCIATION; DEFINITION; FIBROSIS; RISK; MANAGEMENT	63	2020	Gastroenterology & Hepatology	Science Citation Index Expanded (SCI-EXPANDED)	Green Published, gold	WOS:000648939500012
J	Winett, RA; Williams, DM; Davy, BM	Initiating and maintaining resistance training in older adults: a social cognitive theory-based approach	BRITISH JOURNAL OF SPORTS MEDICINE	English	Review		ALL-CAUSE MORTALITY; PHYSICAL-ACTIVITY; CARDIORESPIRATORY FITNESS; GLYCEMIC CONTROL; BREAST-CANCER; WEIGHT-LOSS; EXERCISE; HEALTH; MAINTENANCE; INTERVENTION	60	2009	Sport Sciences	Science Citation Index Expanded (SCI-EXPANDED); Social Science Citation Index (SSCI)	Green Accepted	WOS:000263178000012
J	Guinan, EM; Doyle, SL; Bennett, AE; O'Neill, L; Gannon, J; Elliott, JA; O'Sullivan, J; Reynolds, JV; Hussey, J	Sarcopenia during neoadjuvant therapy for oesophageal cancer: characterising the impact on muscle strength and physical performance	SUPPORTIVE CARE IN CANCER	English	Article	Sarcopenia; Physical fitness; Oesophageal cancer; Neoadjuvant therapy	QUALITY-OF-LIFE; ESOPHAGOGASTRIC CANCER; INTRAABDOMINAL SURGERY; RESISTANCE EXERCISE; BODY-COMPOSITION; CONTROLLED-TRIAL; RATING-SCALE; CHEMOTHERAPY; GUIDELINES; OUTCOMES	52	2018	Oncology; Health Care Sciences & Services; Rehabilitation	Science Citation Index Expanded (SCI-EXPANDED)		WOS:000428813600026
J	Verzola, D; Barisione, C; Picciotto, D; Garibotto, G; Koppe, L	Emerging role of myostatin and its inhibition in the setting of chronic kidney disease	KIDNEY INTERNATIONAL	English	Review	chronic kidney disease; inflammation; insulin resistance; nutrition	IMPROVES INSULIN SENSITIVITY; SKELETAL-MUSCLE MASS; MESSENGER-RNA; MYOBLAST DIFFERENTIATION; AORTIC ATHEROSCLEROSIS; ANTIBODY LY2495655; CANCER CACHEXIA; ADIPOSE-TISSUE; IGF-I; PROTEIN	51	2019	Urology & Nephrology	Science Citation Index Expanded (SCI-EXPANDED)	Bronze	WOS:000459161300011
J	Poggiogalle, E; Migliaccio, S; Lenzi, A; Donini, LM	Treatment of body composition changes in obese and overweight older adults: insight into the phenotype of sarcopenic obesity	ENDOCRINE	English	Review	Body composition; Sarcopenic obesity; Nutrition; Exercise; Physical activity; Pharmacological treatment	RESTING ENERGY-EXPENDITURE; SKELETAL-MUSCLE MASS; INDUCED WEIGHT-LOSS; FAT-FREE MASS; PHYSICAL-ACTIVITY; WHEY-PROTEIN; POSTMENOPAUSAL WOMEN; SEDENTARY BEHAVIOR; INSULIN-RESISTANCE; TRAINING-PROGRAM	49	2014	Endocrinology & Metabolism	Science Citation Index Expanded (SCI-EXPANDED)		WOS:000345404900007
J	Brown, JC; Meyerhardt, JA	Obesity and Energy Balance in GI Cancer	JOURNAL OF CLINICAL ONCOLOGY	English	Review		BODY-MASS INDEX; TREATMENT-RELATED TOXICITY; VISCERAL FAT ACCUMULATION; COLORECTAL-CANCER; PHYSICAL-ACTIVITY; AMERICAN SOCIETY; CLINICAL-TRIALS; RECTAL-CANCER; COLON-CANCER; WEIGHT-LOSS	48	2016	Oncology	Science Citation Index Expanded (SCI-EXPANDED)	Green Accepted, Green Published	WOS:000388931200003
J	Antoun, S; Raynard, B	Muscle protein anabolism in advanced cancer patients: response to protein and amino acids support, and to physical activity	ANNALS OF ONCOLOGY	English	Article	muscle; protein anabolism; sarcopenia; cachexia; physical activity	RANDOMIZED CONTROLLED-TRIAL; CELL LUNG-CANCER; LEAN BODY-MASS; COLORECTAL-CANCER; AEROBIC EXERCISE; PALLIATIVE CARE; RESISTANCE EXERCISE; SARCOPENIC OBESITY;	44	2018	Oncology	Science Citation Index Expanded (SCI-EXPANDED)	Bronze	WOS:000426813100002

							ADIPOSE-TISSUE; CACHEXIA						
J	Takamori, S; Toyokawa, G; Okamoto, T; Shimokawa, M; Kinoshita, F; Kozuma, Y; Matsubara, T; Haratake, N; Akamine, T; Takada, K; Katsura, M; Hirai, F; Shoji, F; Tagawa, T; Oda, Y; Honda, H; Maehara, Y	Clinical Impact and Risk Factors for Skeletal Muscle Loss After Complete Resection of Early Non-small Cell Lung Cancer	ANNALS OF SURGICAL ONCOLOGY	English	Article		DWELLING OLDER-ADULTS; KOREA NATIONAL-HEALTH; PHYSICAL-ACTIVITY; SARCOPENIC OBESITY; EXERCISE; ASSOCIATION; NUTRITION; SURVIVAL; MASS; CHEMOTHERAPY	43	2018	Oncology; Surgery	Science Citation Index Expanded (SCI-EXPANDED)		WOS:000429536700022
S	Kramer, A	An Overview of the Beneficial Effects of Exercise on Health and Performance	PHYSICAL EXERCISE FOR HUMAN HEALTH	English	Article; Book Chapter	Physical activity; Physical performance; Exercise; Medicine; Public health	BONE-MINERAL DENSITY; HIGH-IMPACT EXERCISE; RANDOMIZED CONTROLLED-TRIAL; CROSS-SECTIONAL AREA; PHYSICAL-ACTIVITY; HIGH-INTENSITY; METABOLIC SYNDROME; MENTAL-HEALTH; RESISTANCE EXERCISE; RISK-FACTORS	40	2020	Medicine, Research & Experimental; Physiology; Sport Sciences	Book Citation Index – Science (BKCI-S); Science Citation Index Expanded (SCI-EXPANDED)		WOS:000596720900001
J	Looijaard, SMLM; Slee-Valentijn, MS; Otten, RHJ; Maier, AB	Physical and Nutritional Prehabilitation in Older Patients With Colorectal Carcinoma: A Systematic Review	JOURNAL OF GERIATRIC PHYSICAL THERAPY	English	Review	aged; colorectal neoplasms; exercise therapy; nutrition therapy; prehabilitation	POSTOPERATIVE PULMONARY COMPLICATIONS; HIP FRACTURE PATIENTS; FRAIL ELDERLY-PEOPLE; PROTEIN SUPPLEMENTATION; DOUBLE-BLIND; ENHANCED RECOVERY; CONTROLLED-TRIAL; CANCER SURGERY; COLON-CANCER; MUSCLE MASS	39	2018	Geriatrics & Gerontology; Rehabilitation	Science Citation Index Expanded (SCI-EXPANDED)	Green Published, Bronze	WOS:000450885400007
J	Hojan, K; Milecki, P; Molinska-Glura, M; Roszak, A; Leszczynski, P	Effect of physical activity on bone strength and body composition in breast cancer premenopausal women during endocrine therapy	EUROPEAN JOURNAL OF PHYSICAL AND REHABILITATION MEDICINE	English	Article	Neoplasm; Exercises; Hormones; Osteoporosis; Obesity	MINERAL DENSITY; LEAN MASS; VISCERAL FAT; POSTMENOPAUSAL WOMEN; FEMORAL-NECK; RISK-FACTORS; EXERCISE; SURVIVORS; MUSCLE; TISSUE	39	2013	Rehabilitation	Science Citation Index Expanded (SCI-EXPANDED)		WOS:000323095500007
J	Nieman, DC	Clinical implications of exercise immunology	JOURNAL OF SPORT AND HEALTH SCIENCE	English	Review	Inflammation; Natural killer cells; Neutrophils; Physical activity; Upper respiratory tract infection	C-REACTIVE PROTEIN; RESPIRATORY-TRACT INFECTION; PHYSICAL-ACTIVITY; MODERATE EXERCISE; WEIGHT-LOSS; INFLAMMATORY MARKERS; IMMUNE-RESPONSE; ANTIBODY-RESPONSE; AEROBIC EXERCISE; BODY-COMPOSITION	37	2012	Hospitality, Leisure, Sport & Tourism; Sport Sciences	Science Citation Index Expanded (SCI-EXPANDED); Social Science Citation Index (SSCI)	Green Published, gold	WOS:000208836200006

AIMS Press

© 2025 the Author(s), licensee AIMS Press. This is an open access article distributed under the terms of the Creative Commons Attribution License (https://creativecommons.org/licenses/by/4.0)
